# ALKBH5-mediated m^6^A demethylation fuels cutaneous wound re-epithelialization by enhancing PELI2 mRNA stability

**DOI:** 10.1186/s41232-023-00288-0

**Published:** 2023-07-14

**Authors:** Xin Huang, Yixuan Zhao, Daiming Liu, Shuchen Gu, Yunhan Liu, Yimin Khoong, Shenying Luo, Zewei Zhang, Wenzheng Xia, Meng Wang, Hsin Liang, Minxiong Li, Qingfeng Li, Tao Zan

**Affiliations:** 1 Department of Plastic and Reconstructive Surgery, Shanghai Ninth People’s Hospital, Shanghai JiaoTong University School of Medicine, 639 Zhizaoju Road, Shanghai, 200011 People’s Republic of China; 2 Department of Wound Repair, the Second Affiliated Hospital of Hunan University of Traditional Chinese Medicine, Hunan, China

**Keywords:** *N*^6^-Methyladenosine (m^6^a) RNA modification, ALKBH5, Re-epithelialization, PELI2, RNA stability

## Abstract

**Background:**

Impaired wound re-epithelialization contributes to cutaneous barrier reconstruction dysfunction. Recently, *N*^6^-methyladenosine (m^6^A) RNA modification has been shown to participate in the determination of RNA fate, and its aberration triggers the pathogenesis of numerous diseases. Howbeit, the function of m^6^A in wound re-epithelialization remains enigmatic.

**Methods:**

*Alkbh5*^‒/‒^ mouse was constructed to study the rate of wound re-epithelialization after ALKBH5 ablation. Integrated high-throughput analysis combining methylated RNA immunoprecipitation sequencing (MeRIP-seq) and RNA-seq was used to identify the downstream target of ALKBH5. In vitro and in vivo rescue experiments were conducted to verify the role of the downstream target on the functional phenotype of ALKBH5-deficient cells or animals. Furthermore, the interacting reader protein and regulatory mechanisms were determined through RIP-qPCR, RNA pull–down, and RNA stability assays.

**Results:**

ALKBH5 was specifically upregulated in the wound edge epidermis. Ablation of ALKBH5 suppressed keratinocyte migration and resulted in delayed wound re-epithelialization in *Alkbh5*^‒/‒^ mouse. Integrated high-throughput analysis revealed that PELI2, an E3 ubiquitin protein ligase*,* serves as the downstream target of ALKBH5. Concordantly, exogenous PELI2 supplementation partially rescued keratinocyte migration and accelerated re-epithelialization in ALKBH5-deficient cells, both in vitro and in vivo. In terms of its mechanism, ALKBH5 promoted PELI2 expression by removing the m^6^A modification from PELI2 mRNA and enhancing its stability in a YTHDF2-dependent manner.

**Conclusions:**

This study identifies ALKBH5 as an endogenous accelerator of wound re-epithelialization, thereby benefiting the development of a reprogrammed m^6^A targeted therapy for refractory wounds.

**Graphical Abstract:**

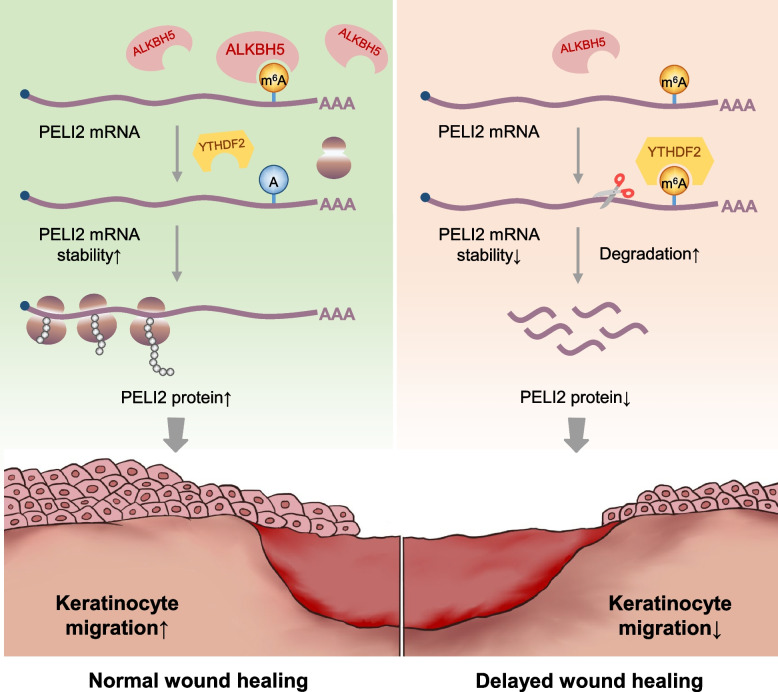

**Supplementary Information:**

The online version contains supplementary material available at 10.1186/s41232-023-00288-0.

## Background

In mammals, the barrier integrity of injured skin is restored through a fundamental mechanism named wound healing, which is a multistep process that involves clotting, inflammation, re-epithelialization, granulation tissue formation, and scar remodeling [1]. Re-epithelialization is considered to be an essential factor that defines successful wound healing; during this process, keratinocytes proliferate and migrate to the wound edge and form a new epidermal sheet that is composed of multiple layers of keratinocytes [2]. Impaired re-epithelialization is the cause of all types of nonhealing chronic wounds that result from trauma, diabetes, vascular disease, infection, or radiation [3]. Chronic wounds have become a substantial challenge for patient families and the healthcare system worldwide; an estimated 1 to 2% of the population will experience chronic wounds during their lifetime, and these wounds are often accompanied by complications such as cellulitis, gangrene, hemorrhage, amputation, and disability [4]. Treating these wound-related complications alone costs the healthcare system in the US over $25 billion each year [5]. Therefore, exploring the mechanisms that regulate wound re-epithelialization is of significant clinical relevance and may provide new insights for therapeutic strategies that accelerate wound healing.

*N*^6^-Methyladenosine (m^6^A) is the most prevalent internal mRNA modification in eukaryotes [6]. In mammals, m^6^A modification is dynamically and reversibly regulated by “writers” (e.g., methyltransferase-like 3 (METTL3), methyltransferase-like 14 (METTL14), and Wilms tumor 1-associated protein (WTAP)) and “erasers” (e.g., fat‒mass and obesity-associated protein (FTO) and α-ketoglutarate-dependent dioxygenase alkB homolog 5 (ALKBH5)) [6–8]. In addition, the m^6^A modification can be recognized by specific RNA-binding proteins that are known as “readers” (YTH domain family 1‒3 (YTHDF1‒3), heterogeneous nuclear ribonucleoprotein (HNRNP), and IGF2 mRNA binding proteins (IGF2BP)). After recognition by “reader” proteins, the m^6^A modification affects various aspects of RNA metabolism including RNA stability, translation, splicing, translocation, and high-level structure [6, 7]. In recent years, various studies have shown that m^6^A modification is widely involved in the maintenance of organ development [9, 10], tissue repair and homeostasis [11, 12], and diseases [6–8, 13].

Recently, a number of studies have demonstrated the extensive involvement of m^6^A modification in the regulation of hair follicle morphogenesis [14], UV-mediated skin injury [15], the development of systemic sclerosis [16], psoriasis vulgaris [17], hypertrophic scarring [18], and tumorigenesis [19–21]. In addition, m^6^A modifications also play important roles in the wound healing process [12]. For example, epidermal-specific ablation of *Mettl14* inhibited wound healing by decreasing the stemness of keratinocytes in vivo [12]. However, whether m^6^A modifications play a role in regulating the migratory ability of keratinocytes remains largely unclear. Moreover, a comprehensive understanding of the role of m^6^A modification in wound re-epithelialization is lacking.

Herein, we identified ALKBH5-mediated m^6^A demethylation as a key regulator of wound re-epithelialization. First, we observed that ALKBH5 is specifically upregulated in keratinocytes at wound edges compared to other m^6^A regulators. Functional analysis showed that ALKBH5 is necessary for cell migration and wound re-epithelialization, as demonstrated by in vitro ALKBH5 knockdown experiments and in vivo cutaneous healing models in *Alkbh5*^‒/‒^ mice. PELI2 was identified as a downstream target of ALKBH5 using high-throughput methylated RNA immunoprecipitation sequencing (MeRIP-seq). Supplementation with exogenous PELI2 partially rescued cell migration in vitro and accelerated wound re-epithelialization in vivo. Furthermore, we found that YTHDF2 specifically binds to the m^6^A site in PELI2 mRNA and promotes its degradation. Overall, this study uncovered a novel mechanism of the epigenetic regulation of wound re-epithelialization by which the m^6^A eraser ALKBH5 facilitates keratinocyte migration by enhancing PELI2 mRNA stability in a YTHDF2-dependent manner.

## Methods

### Patient samples

A total of 10 human chronic wound and adjacent normal skin samples were collected from patients who underwent surgical excision and flap reconstruction in Ninth People’s Hospital, Shanghai Jiaotong University School of Medicine from June 2020 to June 2022 (patient information is summarized in Additional file 1: Table S1), and these samples were used for immunofluorescence staining (IF). Written informed consent was obtained before sample collection in accordance with the Declaration of Helsinki and with approval from the Human Research Ethics Committee of Shanghai Jiao Tong University School of Medicine.

### Cell lines and primary keratinocytes

The HaCaT cell line was purchased from the American Type Culture Collection (Manassas, VA, USA) and confirmed by short tandem repeat (STR) profiling. HaCaT cells were cultured in DMEM (Gibco) supplemented with 10% fetal bovine serum (Gibco) and 1% P/S (Gibco), under standard humid conditions at 37 °C with 5% CO_2_. Primary mouse keratinocytes were isolated from the epidermis of mice (8 weeks of age) after overnight treatment with dispase II (Gibco, 17105041) at 4 °C, followed by digestion with 0.1% trypsin (Gibco, 25300‒054) for 15 min at 37 °C. The keratinocytes were cultured with endothelial cell medium (ECM, ScienCell, 1001).

### Mice and cutaneous wound healing model

All the modeling procedures for establishing the model were conducted in accordance with the Guide for the Care and Use of Laboratory Animals and were approved by the Committee of Animal Care and Use for Research and Education (CACURE) of Shanghai Jiao Tong University School of Medicine. Briefly, the mice were anesthetized, and the backs were shaved. Two full-thickness cutaneous wounds 5 mm in diameter were generated using a standard punch. The fascia below the panniculus carnosus was excised around the wound edge to prevent wound contraction [22]. The wound area was quantified by ImageJ software (National Institutes of Health, Bethesda, MD) based on photos taken on days 0, 2, 4, 6, and 8 after wound establishment. Wound closure was calculated as previously reported: final area/initial area × 100%. The mice were sacrificed at day 8 postwounding. The wound edge and normal skin were excised including complete epithelial margins. The wounds were bisected in the caudocranial direction, and the tissue was either fixed overnight in 4% paraformaldehyde for further histological analysis or immediately frozen in liquid nitrogen for mRNA or protein extraction.

### Generation of Alkbh5^‒/‒^ mice

Male *Alkbh5*^‒/‒^ mice at 6–8 weeks of age were generated via the CRISPR/Cas9 system. Briefly, two sgRNAs-specific for the introns on both sides of the target region of *Alkbh5* were constructed and transcribed in vitro. Cas9 and sgRNA were then microinjected into the zygotes of C57BL/6 J mice. The zygotes were transferred into the oviducts of pseudopregnant ICR females, and F0 mice were born 19–21 days after transplantation. All offspring were identified by PCR analysis and sequencing of DNA from tail samples. A stable F1 generation mouse model was obtained by mating positive F0 generation mice with C57BL/6 J mice. All the mice used in this study were bred and maintained at Gempharmatech Co., Ltd. (Nanjing, Shanghai, China) in accordance with institutional guidelines.

### Plasmid construction and RNA interference

Knockdown of RNA expression in HaCaT cells was achieved by transfection with siRNA sequences synthesized by Zorin Biotechnology Co., Ltd. (Shanghai, China). The sequences of the siRNAs are listed in Additional file 2: Table S2. The PELI2 overexpression cassette was generated by PCR, cloned into the pCDH vector, and verified by DNA sequencing (the sequences of the PELI2 overexpression plasmid are summarized in Additional file 3: Table S3). Transfection of siRNA and overexpression plasmids was performed using Lipofectamine 3000 transfection reagent (Invitrogen, L3000008) according to the manufacturer’s instructions.

### RNA-binding protein immunoprecipitation (RIP)-qPCR

The m^6^A modification or RNA-binding proteins were assessed by RIP experiments using an RNA immunoprecipitation kit (P0101, Geneseed, Shanghai, China) following the manufacturer’s instructions. Briefly, 1.0 × 10^7^ cells were treated with 1 ml RIP lysis buffer. The resulting supernatants were divided into two fractions: 100 μl was kept as input and 900 μl was incubated with specific antibody- or rabbit IgG-conjugated protein A/G magnetic beads in IP buffer supplemented with RNase inhibitors at 4 °C overnight. The immunoprecipitated RNA was digested, purified, and further analyzed by qPCR. The antibodies and primers used for the RIP-qPCR experiment are listed in Additional file 4: Tables S4 and Additional file 5: Table S5, respectively.

### RNA pull‒down

RNA‒protein pull‒down assays were performed using a PureBinding™ RNA‒protein pull‒down kit (P0201, Geneseed, Shanghai, China) according to the manufacturer’s instructions. Biotin-labeled ssRNA probes were synthesized in vitro by Sangon Biotech (Shanghai) Co., Ltd. (the sequences of the ssRNA probes are listed in Additional file 6: Table S6). Briefly, the cell pellets were resuspended and homogenized using 1 ml of standard lysis buffer. Five percent of each sample was used as input. Then, 100 pmol of RNA probes and 50 μl of magnetic beads were incubated with each sample at 4℃ for 1 h with rotation. The eluted protein and input samples were diluted using SDS‒PAGE loading buffer and analyzed by WB.

### Dot-blotting (DB) assay

Total RNA was extracted from tissues according to the protocol used in our previous study [23]. RNA was quantified using a NanoDrop, and 1 μg or 2 μg of RNA was spotted onto a nylon membrane (Millipore, INYC00010). The membranes were cross-linked under ultraviolet (UV) light and then blocked with 5% milk for 1 h at room temperature. The membranes were incubated with an anti-m^6^A antibody (see detailed information in Additional file 4: Tables S4) at 4 °C overnight. The membranes were incubated with horseradish peroxidase (HRP)-conjugated secondary antibodies for 1 h at room temperature and visualized using ECL chemiluminescence (Millipore, WBKLS0100). The same amount of mRNA was spotted on the membranes and stained with 0.02% methylene blue as a loading control.

### MeRIP-seq

MeRIP-seq was performed as described previously [24, 25]. In brief, total RNA was extracted from human epidermis samples (see sample details in Additional file 7: Table S7) and HaCaT cells and fragmented into approximately 100-nucleotide-long fragments. Approximately 5% fragmented RNA was used as input, and the remaining RNA was enriched by immunoprecipitation using affinity-purified anti-m^6^A polyclonal antibodies (ABE572, Millipore, Germany). Sequencing was carried out using an Illumina NovaSeq 6000 platform. The MeRIP-seq data were deposited in the GEO database (GSE 211442).

### RNA-seq and data analysis

Total RNA was extracted from HaCaT cells using TRIzol reagent (Invitrogen, Carlsbad, CA, USA). Poly-T oligo-attached magnetic beads were used to enrich eukaryotic mRNA. After fragmentation, mRNA was converted into individual cDNA libraries. After cluster generation, the library preparations were sequenced on an Illumina Novaseq™ 6000 platform. Gene expression levels were quantified by fragments per kilobase of exon model per million mapped reads (FPKM). The DESeq2 algorithm was used to identify differentially expressed genes, and a false discovery rate (FDR) < 0.05 and | log2(fold change) |≥ 1 were used as the thresholds. The RNA-seq data were deposited in the GEO database (GSE 211076).

Gene otology (GO) analysis of designated genes was performed using DAVID (http://david.abcc.ncifcrf.gov/). Fisher’s exact test was used to identify the significant GO categories, and FDR was used to correct the *P* values. GO terms with *P* < 0.05 were considered to be statistically significant. Enrichment maps were created using Cytoscape 3.7.0, and bubble plots were constructed using Prism GraphPad 9.0 (GraphPad Software, Inc.). The correlation network of PELI2 with other candidate genes or with cell migration was constructed using IPA (Ingenuity Systems).

### In vivo* cell tracking*

HaCaT cells were seeded in 12-well plates at 40% cell density one day before siRNA or overexpression plasmid transfection. The attached cells were scratched with a 200-μl pipette tip when they grew to confluence. The cells were then subjected to phase contrast time-lapse imaging experiments using an automated inverted microscope (IX‒81, Olympus, Tokyo, Japan) equipped with an incubator that regulated temperature (37℃), humidity (95% relative humidity), and CO_2_ concentration (5%). High-resolution images of selected fields were captured successively every 2 h for 38 h. The exported video data were analyzed using PIVlab software (Version 2.56) for MATLAB (R2021a) following the software instructions [26].

### Cell proliferation assay

Cell proliferation was assessed by a CCK-8 kit (Dajindo, Japan) and Ed-U DNA Cell Proliferation kit (Beyotime Biotechnology, China) following the manufacturer’s instructions, as we previously described in a previous study [27].

### Cell apoptosis and cell cycle assay

Cell apoptosis was assessed as we described in a previous study using a FITC-Annexin V apoptosis kit (BD Biosciences, San Diego, CA) following the manufacturer’s instructions [23]. Briefly, the cells were washed twice with cold PBS and stained with FITC-Annexin V and PI on ice for 5 min. For cell cycle analysis, the cells were collected and fixed with 75% cold ethanol at 4 °C for 2 h. Cells were rinsed with PBS 3 times and stained with RNase A and propidium iodide (Cell Cycle Assay Kit, Dojindo, Japan) successively according to the manufacturer’s instructions. The samples were subjected to flow cytometry analysis (BD LSRFortessa analyzer, BD Biosciences).

### Transwell assay

A 24-well Transwell system with polycarbonate filters (8-μm pores, Corning, USA) was used for the Transwell assay according to the protocol described in our previous study [28]. The area of cells that migrated from the membrane was analyzed by ImageJ software.

### Wound healing assay

HaCaT cells were seeded in 6-well plates and transfected with siRNAs or overexpression plasmids. After reaching confluence, the attached cells were scratched with a 200-μl pipette tip. The cells were then cultured at 37℃ in 5% CO_2_, and images were captured at 0 h, 24 h, 48 h, and 72 h using an inverted microscope (Nikon, Japan). The wound areas were measured with ImageJ software and normalized to the wound area at 0 h.

### Quantitative real-time PCR

Total RNA was extracted using TRIzol reagent (Invitrogen). Complementary DNA was synthesized using 1000 ng RNA with PrimeScript RT Master Mix (Takara, RR036A). qRT-PCR was performed on an ABI QuantStudio 6 Flex system using SYBR Premix (Takara, RR066A) according to the manufacturer’s instructions. The sequences of the primers are summarized in Additional file 5: Table S5.

### WB and IF

WB and IF staining were performed according to the protocol described in our previous study [23]. The antibodies used for WB and IF are listed in Additional file 4: Table S4. The uncropped original western blots in this article are provided in Additional file 8: Fig. S1.

### Statistical analysis

GraphPad Prism 9 was used for the statistical analyses. The values are presented as the mean ± SD, and statistical significance was determined as indicated in each figure legend. *P < 0.05, **P < 0.01, ***P < 0.001, ****P < 0.0001. The distribution of data was first examined using the Shapiro–Wilk normality test, Kolmogorov–Smirnov test, and D’Agostino & Pearson normality test. The comparison between two groups was conducted using a two-tailed unpaired Student’s *t* test if the data fit a normal distribution and variances were similar by *F* test (*P* > 0.05). For variances that differed according to the *F* test (*P* < 0.05), two-tailed unpaired Student’s *t* test with Welch’s correction was used. The Mann–Whitney *U* test was conducted when the data did not fit a normal distribution. If the variance among three or more groups was minimal, ANOVA followed by Dunnett’s posttest or Turkey’s post-hoc test was used for multigroup comparisons.

## Results

### Decreased RNA m^6^A modification and increased ALKBH5 expression were observed in keratinocytes at wound edges>

**Fig. 1:**
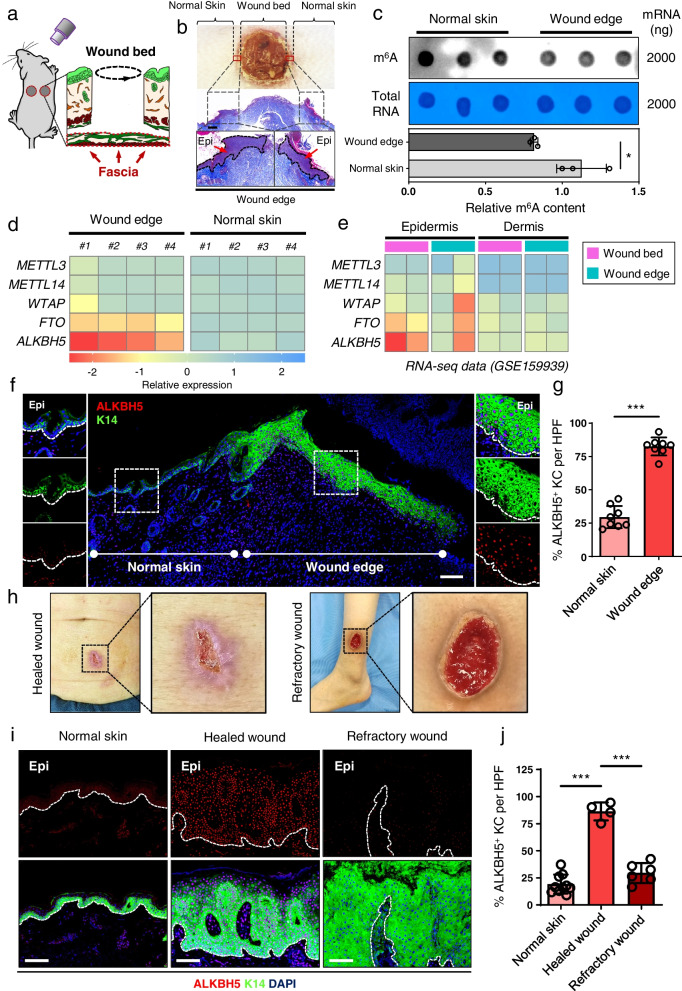
Decreased RNA m^6^A modification and increased ALKBH5 expression in keratinocytes in healthy wound edges. **a** Generation of 5-mm diameter full-thickness skin wounds on mouse backs with the fascia (red dashed lines) below the panniculus carnosus excised to prevent wound contraction. **b** The gross appearance and histology of skin wounds at post wound day 2 (PWD2). The wound bed, wound edge, and adjacent normal skin were outlined and designated. Scale bar: 250 μm. **c** Global m^6^A levels of mRNA extracted from the skin of wound edges and adjacent normal skin were measured by m^6^A DB assays. Total RNA was determined by methylene blue staining (as a loading control). The experiments were performed in triplicate. *t* test, two-sided *P < 0.05. **d** Heatmap of m^6^A “writer” and “eraser” gene expression in wound edges and normal skin. The experiments were performed with four biological replicates. **e** Heatmap of m^6^A “writer” and “eraser” gene expression in the epidermis and dermis of the wound bed and wound edge according to RNA-seq data (GSE159939). **f** ALKBH5 expression in the skin of the wound edge was visualized by IF. Scale bar: 100 μm. **g** Statistical analysis of the percentage of ALKBH5^+^ keratinocytes per HPF. Wound samples were collected from four mice, and the percentage of ALKBH5^+^ cells is shown as the mean ± SD. *t* test, two-sided ***P < 0.001. **h** Representative clinical photography showing a cohort of patients with chronic wounds characterized by healed or refractory nonhealing wounds. **i** ALKBH5 expression in the wound edges of healed wounds, refractory wounds, and normal skin was visualized by IF. Scale bar: 100 μm. **j** Statistical analysis of the percentage of ALKBH5^+^ epidermal cells per HPF. The wound edges of four healed wounds, six refractory wounds, and their corresponding normal skin controls were analyzed. The percentage of ALKBH5^+^ cells is shown as the mean ± SD. One-way ANOVA, ***P < 0.001. Epi epidermis. Dotted lines denote epidermal boundaries

To investigate the functional role of RNA m^6^A modifications in wound re-epithelialization, we generated full-thickness skin wounds on the backs of mice (Fig. 1a and b). We observed a significant decrease in global m^6^A modification in the skin at the wound edge compared to adjacent normal skin using anti-m^6^A dot-blotting (DB) (Fig. 1c). Furthermore, we measured the expression levels of m^6^A “writers” and “erasers,” and we observed a significant upregulation of ALKBH5 and FTO mRNA expression in the skin of wound edges, while the expression of other regulators remained unchanged (Fig. 1d). Consistently, this finding was further confirmed by previous transcriptomic analysis (GSE159939) [29], which highlighted the elevated mRNA expression of ALKBH5 and FTO in the epidermis of the wound edge (Fig. 1e). Immunofluorescence staining showed that ALKBH5 is expressed at a relatively low level in normal skin, including the epidermis, skin appendages, and some dermal cells (Additional file 9: Fig. S2). Importantly, an increased percentage of ALKBH5^+^ keratinocytes (identified by Keratin 14 staining) was observed in the epidermis at the wound edge (Fig. 1f, g). Additionally, ALKBH5^+^ cells also showed significant accumulation in the dermis at the wound edge (Additional file 9: Fig. S2), suggesting that ALKBH5 is also involved in dermal reconstruction during wound healing. FTO, another m^6^A demethylase, also showed similar upregulation as ALKBH5 in both keratinocytes and fibroblasts at the wound edge when compared to normal skin (Additional file 10: Fig. S3). However, ALKBH5 was more upregulated than FTO at the wound edge. In addition, the expression of m^6^A methylase METTL3 and METTL14 remained unchanged in both keratinocytes and fibroblasts at the wound edge when compared to normal skin (Additional file 10: Fig. S3). Collectively, these data suggest that elevated ALKBH5 expression is primarily responsible for decreased m^6^A modification of RNA in keratinocytes at the wound edge.

Interestingly, in a cohort of clinical wounds (patient information is listed in Additional file 1. Table S1), the percentage of ALKBH5^+^ keratinocytes was increased only in the epidermis of healed wounds compared to that of refractory wounds and normal skin (Fig. 1h–j). Since keratinocytes in the wound edge are responsible for the reconstruction of the epidermal barrier, our results suggest that ALKBH5 expression may be relevant to the function of keratinocytes during wound re-epithelialization.

Although our results showed a similar upregulation of FTO in the wound edge epidermis as ALKBH5, we did not observe a significant change in FTO expression at both the mRNA and protein levels in HaCaT upon ALKBH5 knockdown (Additional file 11: Fig. S4a-c). Consistently, the FTO expression in the skin of *Alkbh5*^*‒/‒*^ mice remained unchanged when compared to that of WT mice (Additional file 11: Fig. S4d-f). These results suggest that ALKBH5 could not regulate the expression of FTO and there is negligible compensatory expression of FTO upon ALKBH5 knockdown.

### ALKBH5 inhibition decreases the migration of keratinocytes

**Fig. 2:**
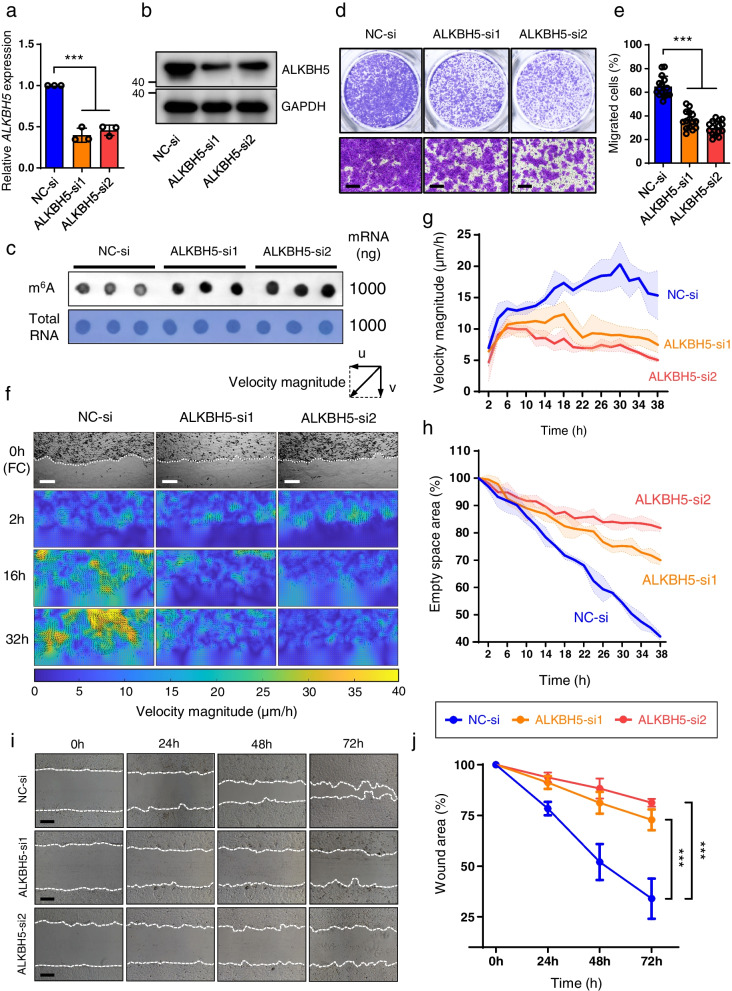
Inhibition of ALKBH5 impairs keratinocyte migration in vitro. **a, b** Decreased ALKBH5 expression was confirmed in HaCaT cells after siRNA transfection by qRT‒PCR (**a**) and WB (**b**). The experiments were performed in triplicate. One-way ANOVA, ***P < 0.001. The full-length blots are presented in Additional file 8: Fig. S1. **c** Global m^6^A levels of mRNA extracted from ALKBH5-knockdown or control HaCaT cells after siRNA transfection were determined by m^6^A DB assays. Total RNA was determined by methylene blue staining (as loading control). **d** Cell migration of ALKBH5-knockdown or control HaCaT cells was analyzed by Transwell assay. Scale bar: 25 μm. **e** Statistical analysis of the Transwell assay. All of the experiments were performed in triplicate, and five random fields were included in the analysis. The percentage of migrated cell areas is shown as the mean ± SD. One-way ANOVA, ***P < 0.001. **f** Representative dynamic cell velocity mapping of ALKBH5-knockdown or control HaCaT cells at 0 h, 2 h, 16 h, and 32 h after scratching using cell tracking imaging. *u* velocity in the *x* direction, *v* velocity in the *y* direction. The velocity magnitude represents the resultant velocity of *u* and *v*. The direction and magnitude of the velocity are indicated with black arrows and color density, respectively. FC phase‒contrast image. Scale bar: 250 μm. **g** Statistical analysis of the velocity magnitude of HaCaT cells in the ALKBH5 knockdown and control groups at different time points. **h** Statistical analysis of the healing rate of scratched wounds. All of the experiments were performed in triplicate. **i** The long-term migratory ability of HaCaT cells was evaluated with wound scratching assays. Scale bar: 100 μm. **j** Statistical analysis of wound healing assays. All of the experiments were performed in triplicate, and 3 random fields were included at each time point for analysis. One-way ANOVA, ***P < 0.001

To determine the biological functions of ALKBH5 in keratinocytes, we first silenced the expression of ALKBH5 in the human keratinocyte HaCaT cell line using two individual small interfering RNAs (siRNAs) (Fig. 2a and b). Accordingly, we found that the global m^6^A level was increased after ALKBH5 knockdown (Fig. 2c). The reduced cell migratory velocity after ALKBH5 silencing was confirmed by using Transwell assay (Fig. 2d and e), dynamic cell tracking imaging (Fig. 2f–h) and wound scratching assay (Fig. 2i and j). However, the proliferative ability of HaCaT cells remained unaltered upon the deprivation of ALKBH5, as demonstrated by CCK-8 (Additional file 12: Fig. S5a), Ed-U (Additional file 12: Fig. S5b and S5c), and flow cytometry analysis (Additional file 12: Fig. S5d and S5e). In addition, the depletion of ALKBH5 did not result in a significant change in the apoptosis rate in keratinocytes (Additional file 12: Fig. S5f and S5g). These results demonstrated that ALKBH5 specifically modulates cellular migration rather than the proliferation and apoptosis of keratinocytes in vitro.

### Ablation of ALKBH5 inhibits wound re-epithelialization in mice

**Fig. 3:**
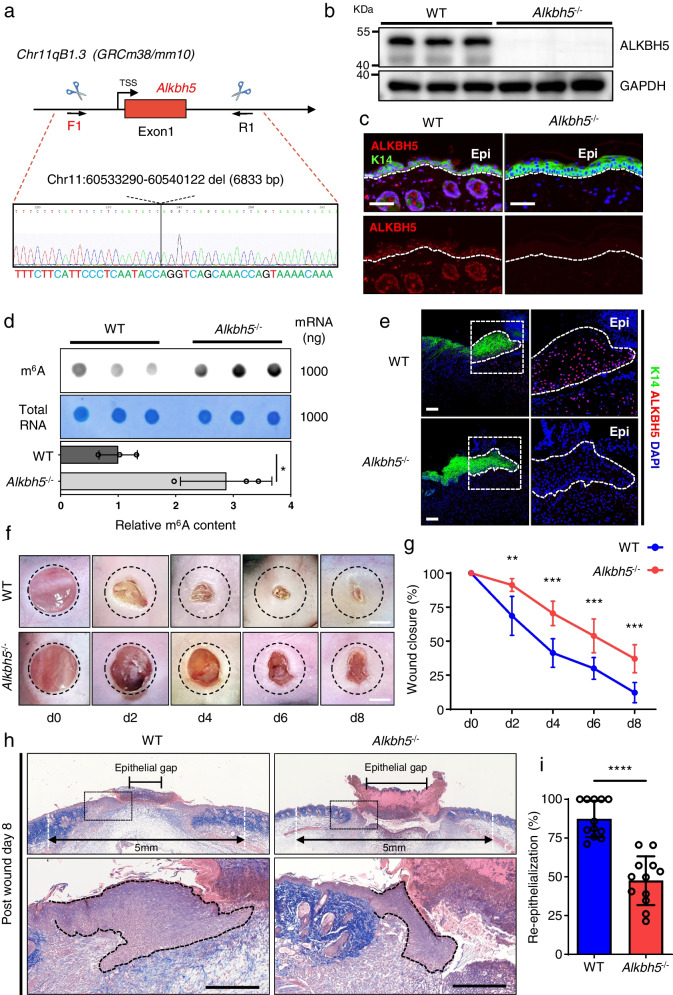
Loss of ALKBH5 impairs wound re-epithelialization in mice. **a** Schematic ideogram showing the strategy for the deletion of the ALKBH5 gene using CRISPR/Cas9 technology. **b, c** The loss of ALKBH5 expression in the skin and epidermis of *Alkbh5*^‒/‒^ mice was confirmed by WB (**b**) and IF (**c**), respectively. The full-length blots are presented in Additional file 8: Fig. S1. **d** The global m^6^A level of skin at the wound edge of WT and *Alkbh5*^‒/‒^ mice was measured by m^6^A DB assays. Total RNA was determined by methylene blue staining (as a loading control). The experiments were performed in triplicate, and the relative m^6^A modification contents are shown as the mean ± SD. *t* test, two-sided *P < 0.05. **e** IF revealed the loss of ALKBH5 expression in the epidermis at the wound edge of *Alkbh5*^‒/‒^ mice. Scale bar: 100 μm. Dotted lines denote epidermal boundaries. Epi epidermis. **f, g** Representative images of cutaneous wounds (**f**) of WT and *Alkbh5*^‒/‒^ mice on PWD0, PWD2, PWD4, PWD6, and PWD8. Scale bar: 2 mm. The black dashed circle delineates the original wound of 5 mm width. Rates of wound closure (**g**) were quantified by using ImageJ software and are expressed as the percentage of the nonhealing area. Twelve wounds of six mice were included in the analysis. The relative percentages of wound closure are shown as the mean ± SD. *t* test, two-sided **P < 0.01, ***P < 0.001. **h**, **i** Histological analysis (**h**) of wound re-epithelialization in WT and *Alkbh5*^‒/‒^ mice at PWD8. The line with arrowheads indicates the 5 mm width of the original wound gap. The line with blunt ends delineates the epithelial gap, which represents the nonepithelialized wound area. Scale bar: 1 mm. The percentage of re-epithelialization was quantified (**i**) in WT and *Alkbh5*^‒/‒^ mice at PWD8. Twelve wounds of six mice were included in the analysis. The relative percentages of re-epithelialized wound area are shown as the mean ± SD. Mann–Whitney test, two-sided ****P < 0.0001. Scale bar: 1 mm. Dotted lines denote epidermal boundaries

Based on the regulatory role of ALKBH5 in the cellular function of keratinocytes in vitro, we next investigated the role of ALKBH5 in wound re-epithelialization in vivo using an *Alkbh5-*knockout (*Alkbh5*^‒/‒^) mouse model (Fig. 3a). The *Alkbh5*^‒/‒^ mice were similar to their WT littermates in terms of gross skin appearance and hair growth. Skin histology showed no difference in the thickness of the epidermis after ALKBH5 deletion; however, a degenerated and thinned intradermal adipose tissue layer as well as compensatory thicker dermis were observed (Additional file 13: Fig. S6). Immunoblotting and IF staining confirmed the loss of ALKBH5 expression throughout the skin layers of *Alkbh5*^‒/‒^ mice (Fig. 3b and c).

Full-thickness skin wounds were generated on the backs of mice to induce the proliferation and migration of quiescent keratinocytes. The increase in global m^6^A modification and diminished ALKBH5 expression in the skin of the wound edge of *Alkbh5*^‒/‒^ mice were confirmed by DB (Fig. 3d) and IF staining (Fig. 3e), respectively. During the healing process, *Alkbh5*^‒/‒^ mice exhibited a significant delay in wound closure compared to WT mice (Fig. 3f and g). Histological analysis further revealed a decreased re‒epithelialization rate with an enlarged epithelial gap in *Alkbh5*^‒/‒^ mice (Fig. 3h and i). Consistently, we did not observe a significant change in Ki67 expression in the epidermis at the wound edge of *Alkbh5*^‒/‒^ mice, which further indicates that Alkbh5 specifically regulates the migration of keratinocytes (Additional file 14: Fig. S7). Together, our results suggest that m^6^A modification mediated by ALKBH5 plays a vital role in wound re-epithelialization in vivo.

### Genome-wide MeRIP-seq and RNA-seq identified PELI2 as the downstream target of ALKBH5

**Fig. 4:**
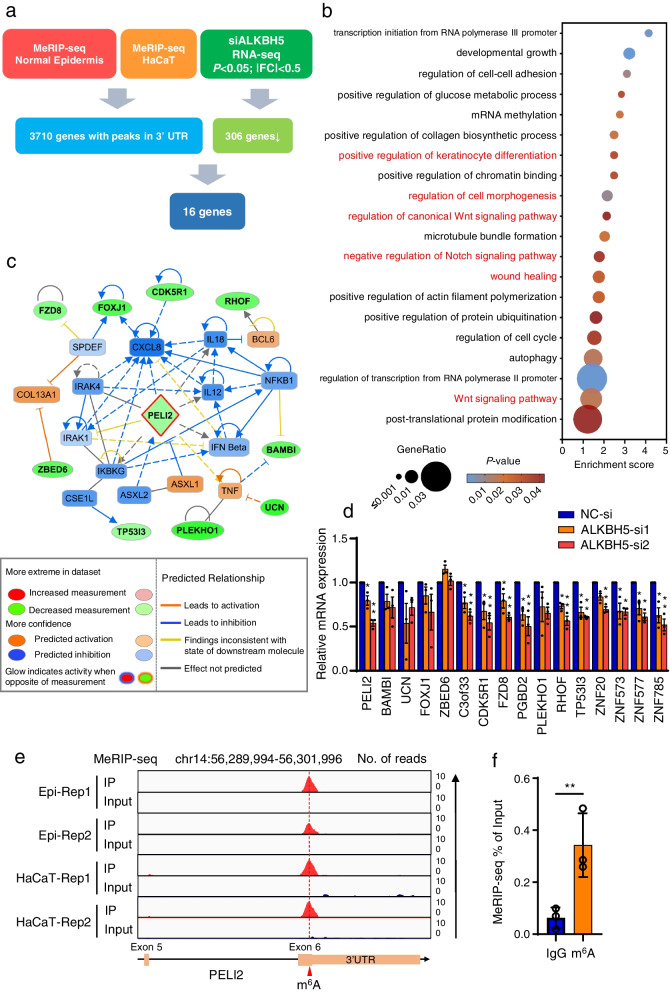
Characterization of m^6^A‒modified genes that are regulated by ALKBH5 in human epidermis samples and keratinocyte cell lines. **a** Flowchart of m^6^A modification downstream analysis. MeRIP-seq identified 3710 genes shared by human epidermis samples and keratinocyte cell lines, with specific m^6^A peaks in the 3’UTR. RNA-seq identified 306 downregulated genes after ALKBH5 knockdown; the cutoffs used to define downregulated genes were |FC|< 0.5 and *P* < 0.05. FC fold change. Finally, 16 candidate genes with both m^6^A peaks and differential expression were identified as potential downstream targets of ALKBH5. **b** GO enrichment map of 3710 genes. **c** The regulatory network of PELI2 and 15 other candidate genes generated by IPA. Please note that *C3orf33*, *PGBD2*, *ZNF20*, *ZNF573*, *ZNF577*, and *ZNF785* are not shown as they have no pathway connections with PELI2. **d** The expression of 16 candidate genes after ALKBH5 knockdown was measured using qRT‒PCR. The experiments were performed in triplicate, and the relative mRNA expression is shown as the mean ± SD. One-way ANOVA, *P < 0.05; **P < 0.01. **e** IGV tracks displaying the MeRIP-seq read coverage of PELI2 in human epidermis and HaCaT. **f** m^6^A-RIP-qPCR assays confirmed the m^6^A modification of PELI2 transcripts. The experiments were performed in triplicate. The relative mRNA expression in the anti-m^6^A antibody group was compared to that in the IgG group, and it is shown as the mean ± SD. One-way ANOVA, **P < 0.01

To understand the regulatory role of m^6^A modifications in keratinocytes, we mapped the m^6^A sites in normal human epidermal tissue and keratinocyte cell lines by methylated RNA immunoprecipitation sequencing (MeRIP-seq) (Fig. 4a). MeRIP-seq identified ~ 20,000 m^6^A peaks in each sample (Additional file 15: Table S8). These m^6^A peaks were mostly enriched in the 3’UTRs, especially near stop codons (Additional file 16: Fig. S8a and S8b). The typical motifs of these m^6^A modifications were characterized by the canonical RRACH (R = G or A; H = A, C or U) sequence as reported by previous studies (Additional file 16: Fig. S8c) [30]. We then identified 3710 genes shared in all tested samples and with m^6^A peaks in the 3’UTRs of the corresponding mRNAs (Additional file 16: Fig. S8d and S8e). Interestingly, the gene ontology analysis revealed a close association of these m^6^A-modified genes with the regulation of epidermal development and homeostasis, as these genes were enriched in pathways including regulation of keratinocyte differentiation, cell morphogenesis, Notch signaling pathway, Wnt signaling pathway, and wound healing (Fig. 4b; Additional file 17: Fig. S9).

As the knockdown of demethylase ALKBH5 would increase the m^6^A modification and facilitate the interaction with YT521-B homology domain-containing Family (YTHDF) proteins, which are major m^6^A reader proteins and responsible for RNA degradation [31]. As a result, we mainly focused on the down-regulated genes upon ALKBH5 knockdown. By combining RNA-seq and previously characterized m^6^A-modified genes, we identified 16 candidate genes whose expression may be associated with the level of ALKBH5-regulated m^6^A modification (Fig. 4a). Functional enrichment revealed that these genes may play a role in the regulation of epithelium development, cellular functions in terms of epithelial cell differentiation, cell division, cell migration, cytoskeleton organization, and signaling pathways including Wnt signaling (Additional file 18: Fig. S10). Subsequent ingenuity pathway analysis (IPA) of the 16 genes revealed that Pellino E3 ubiquitin protein ligase family member 2 (PELI2) is located in the core of the candidate gene matrix (Fig. 4c). Furthermore, IPA revealed that PELI2 can modulate the migration of keratinocytes through multiple pathways (Additional file 19: Fig. S11), suggesting that PELI2 may be a downstream effector of ALKBH5-mediated regulation of cellular migration.

A quantitative RT‒PCR assay further confirmed that PELI2 was decreased after ALKBH5 knockdown (*P* < 0.05) (Fig. 4d). MeRIP-seq showed that PELI2 had a m^6^A peak in the last (6th) exon (Fig. 4e). Consistently, MeRIP-qPCR further validated that PELI2 was modified with an abundant m^6^A signal (Fig. 4f). Taken together, these findings indicate that PELI2, as a m^6^A-modified gene, is potentially regulated by ALKBH5.

### ALKBH5 regulates the m^6^A methylation and expression of PELI2

**Fig. 5:**
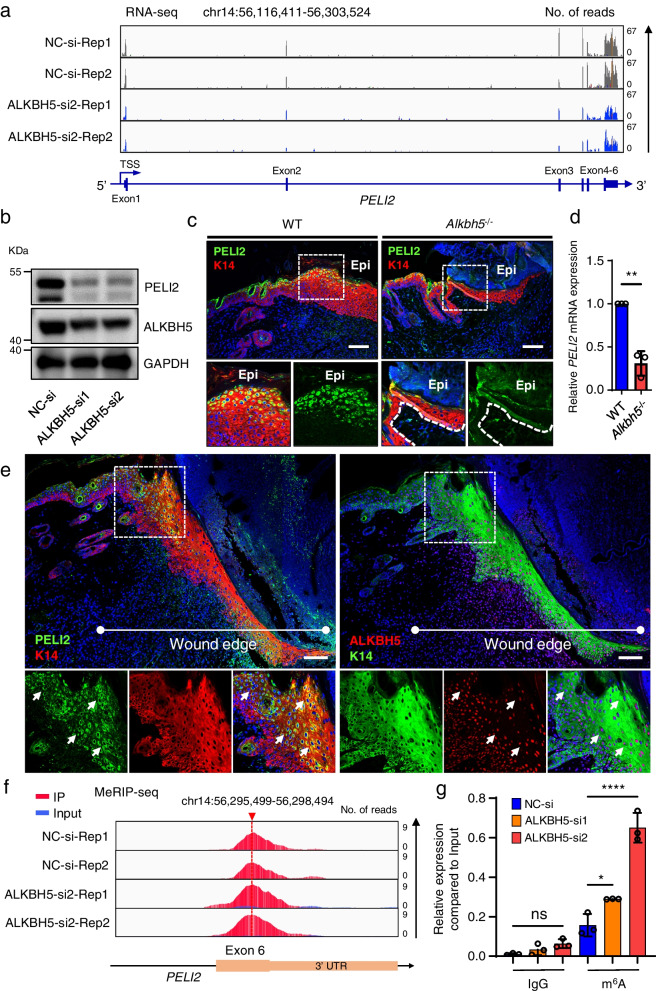
ALKBH5 expression is positively correlated with PELI2 expression levels. **a** IGV tracks of PELI2 expression according to the RNA-seq analysis of ALKBH5-knockdown or control HaCaT cells. The experiments were performed in duplicate. **b** PELI2 protein expression was measured in HaCaT cells after ALKBH5 knockdown by WB. The full-length blots are presented in Additional file 8: Fig. S1. **c** PELI2 expression in the skin at the wound edge of WT and *Alkbh5*^‒/‒^ mice was visualized by IF staining at PWD8. Dotted lines denote epidermal boundaries. Scale bar: 100 μm. **d** qRT-PCR showed the mRNA expression of PELI2 in primary keratinocytes derived from WT and *Alkbh5*^‒/‒^ mice. The experiments were performed in triplicate, and the relative mRNA expression is shown as the mean ± SD. *t* test, two-sided **P < 0.01. **e** The expression and colocalization of PELI2 and ALKBH5 in the epidermis at the wound edge of WT mice were visualized using IF staining at PWD6. Scale bar: 100 μm. **f** IGV tracks displaying the MeRIP-seq read coverage of PELI2 after ALKBH5 knockdown. The experiments were performed in duplicate. **g** Increased m^6^A modification in PELI2 transcripts after ALKBH5 knockdown in HACAT cells, as assessed by gene-specific m^6^A-RIP-qPCR assays. The experiments were performed in triplicate, and the relative expression of mRNA in each group was compared to the input and is shown as the mean ± SD. One-way ANOVA, ns not significant, *P < 0.05, ****P < 0.0001

To further elucidate the role of ALKBH5 in regulating PELI2 expression, we found a significant downregulation of PELI2 expression in ALKBH5-deficient cells, as demonstrated by RNA-seq (Fig. 5a) and western blotting (Fig. 5b). IF showed that PELI2 was not expressed in both the keratinocytes and dermal fibroblasts of normal skin, but was specifically upregulated in suprabasal keratinocytes at the wound edge of WT mice (Additional file 20: Fig. S12). Consistently, decreased PELI2 protein expression was observed in keratinocytes at the wound edge in *Alkbh5*^*‒/‒*^ mice compared to wild-type littermates (Fig. 5c). The primary keratinocytes derived from *Alkbh5*^*‒/‒*^ mice also showed markedly reduced PELI2 expression (Fig. 5d; Additional file 21: Fig. S13). Moreover, colocalization of PELI2 and ALKBH5 could be observed in the epidermis at the wound edge (Fig. 5e). These data support that ALKBH5 positively regulates PELI2 expression, both in vitro and in vivo.

We next investigated whether ALKBH5-mediated PELI2 regulation is associated with m^6^A modification. As expected, enrichment of the m^6^A modification of PELI2 was observed after depleting ALKBH5 in keratinocytes, as revealed by MeRIP-seq (Fig. 5f) and MeRIP-qPCR (Fig. 5g). Collectively, this evidence indicated that ALKBH5 regulates PELI2 expression with a salient change in the m^6^A level.

### Exogenous expression of PELI2 rescues delayed wound re-epithelialization after ALKBH5 depletion

**Fig. 6:**
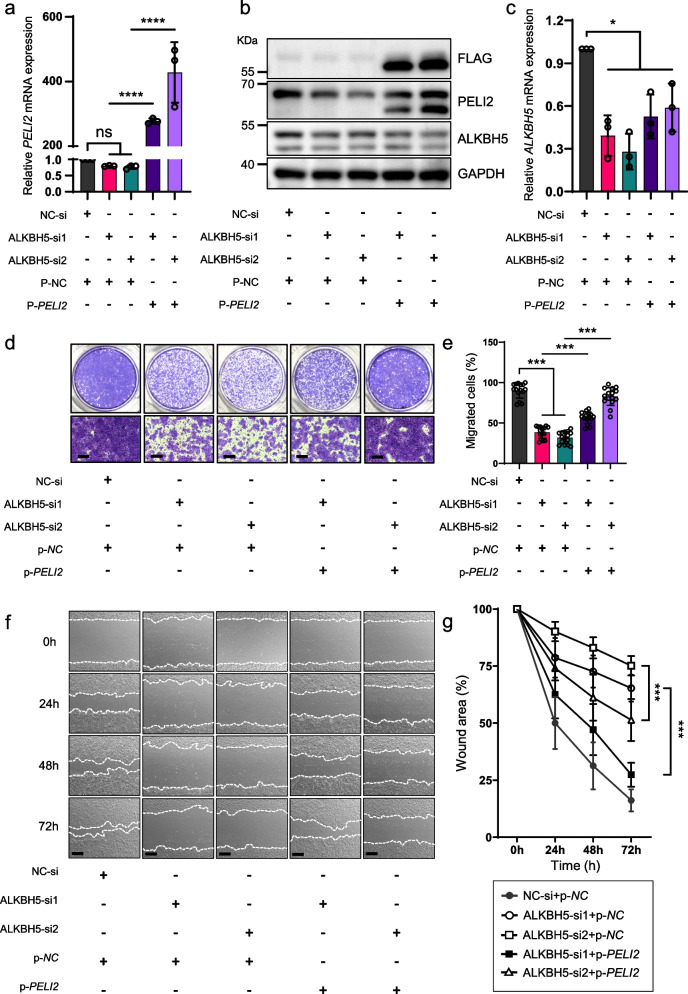
Exogenous PELI2 overexpression rescues the effects of ALKBH5 knockdown on keratinocyte migration. **a, b** qRT‒PCR showing the mRNA expression of PELI2 (**a**) and ALKBH5 (**b**) in HaCaT cells treated with ALKBH5 or NC siRNAs and transfected with PELI2 or NC plasmids for 48 h. The experiments were performed in triplicate, and the relative expression of mRNA is shown as the mean ± SD. One-way ANOVA, ns not significant, *P < 0.05, ****P < 0.0001. The full-length blots are presented in Additional file 8: Fig. S1. **c** The protein expression of ALKBH5 and PELI2 was determined by WB. **d** Transwell assays showed the effect of exogenous PELI2 overexpression on the migration of ALKBH5-knockdown or control HaCaT cells. Scale bar: 25 μm. **e** Statistical analysis of the Transwell assay results. All of the experiments were performed in triplicate, and five random fields were included in the analysis. The percent area of migrated cells is shown as the mean ± SD. One-way ANOVA, ***P < 0.001. Scale bar: 25 μm. **f** The effect of exogenous PELI2 overexpression on the migration of ALKBH5-knockdown or control HaCaT cells was evaluated by wound scratching assay. Scale bar: 100 μm. **g** Statistical analysis of wound scratching assays. All of the experiments were performed in triplicate, and three random fields at each time point were included in the analysis. Mann–Whitney test (at 24 h), no significant difference was found between groups of ALKBH5-si1 + p-*PELI2* vs. ALKBH5-si1 + p-*NC* and ALKBH5-si2 + p-*PELI2* vs. ALKBH5-si2 + p-*NC*. One-way ANOVA (at 48 h, 72 h), ***P < 0.001

Since PELI2 is modulated by ALKBH5, we then explored whether exogenous overexpression of PELI2 could rescue the inhibitory phenotype in ALKBH5-deficient cells. The restored exogenous PELI2 expression was demonstrated by qRT‒PCR (Fig. 6a) and western blotting (labeled by Flag tag, Fig. 6b). Importantly, ALKBH5 expression remained unaltered after reintroducing PELI2, underscoring that PELI2 is the downstream target of ALKBH5 (Fig. 6c).

**Fig. 7:**
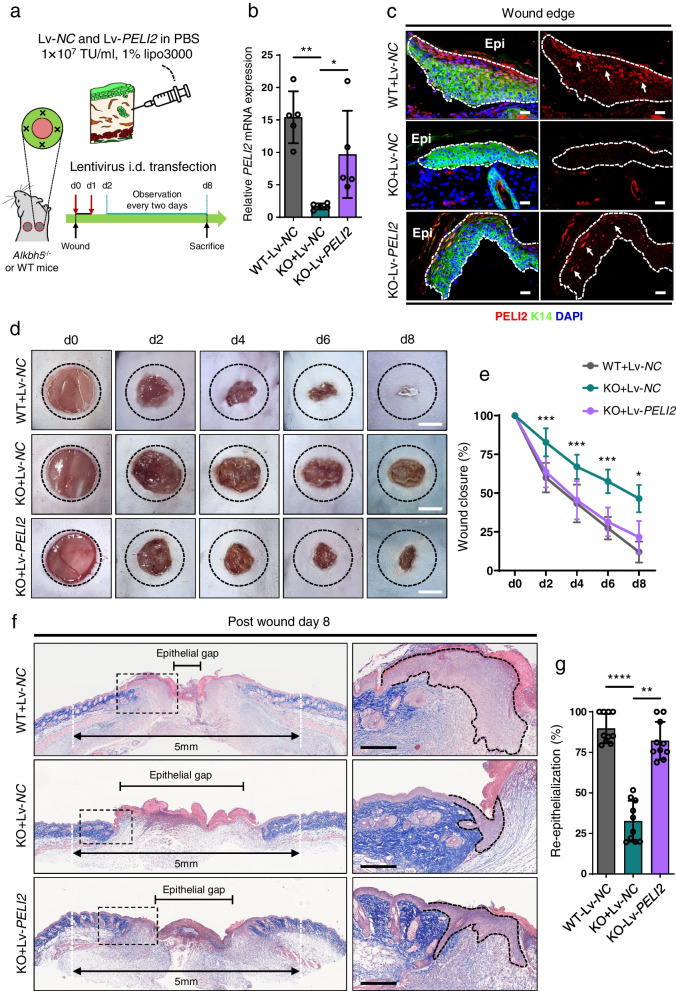
Exogenous PELI2 overexpression rescues the effect of ALKBH5 knockdown on re-epithelialization and wound healing in vivo. **a** Schematic ideogram showing the intradermal injection of Lv-*PELI2* and Lv-*NC* (1 × 10^7^ TU/ml, 1% Lipo3000) into the skin at the wound edge of WT and *Alkbh5*^‒/‒^ mice at d0 and d1 after wound establishment. All the mice were sacrificed at PWD8. **b** The restoration of PELI2 mRNA expression in the skin of the wound edge of *Alkbh5*^‒/‒^ mice was confirmed by qRT‒PCR. Five wounds from five mice were included in the analysis. The relative gene expression levels are shown as the mean ± SD. One-way ANOVA, *P < 0.05, **P < 0.01. **c** IF confirmed the restoration of PELI2 expression in the epidermis of the wound edge of *Alkbh5*^‒/‒^ mice after Lv-*PELI2* transfection. Dotted lines denote epidermal boundaries. Epi epidermis. Scale bar: 25 μm. **d, e** Representative images of cutaneous wounds (**d**) of WT mice transfected with Lv-*NC* and *Alkbh5*^‒/‒^ mice transfected with Lv-*PELI2* or Lv-*NC* on PWD0, PWD2, PWD4, PWD6, and PWD8. Scale bar: 2 mm. The black dashed circle delineates the original wound of 5 mm width. The rate of wound closure (**e**) was quantified by using ImageJ software and is expressed as the percentage of the nonhealing wound area. Ten wounds of five mice were included in the analysis. The relative percentages of wound closure are shown as the mean ± SD. One-way ANOVA (at 2 days, 4 days, 6 days), ***P < 0.001. Mann–Whitney test (at 8 days), two-sided *P < 0.05 (KO + Lv-*PELI2* vs. KO + Lv-*NC*). **f, g** Histological analysis (**f**) of wound re-epithelialization in WT mice transfected with Lv-*NC* and *Alkbh5*^‒/‒^ mice transfected with Lv-*PELI2* or Lv-*NC* at eight days after wounding. The line with arrowheads indicates the 5 mm width of the original wound gap. The line with blunt ends delineates the epithelial gap, indicating the width of the nonepithelialized wound area. Quantification of the percentage of re-epithelialization (**g**). Ten wounds of five mice were included in the analysis. The relative percentages of re-epithelialized wounds are shown as the mean ± SD. Mann–Whitney test, two-sided **P < 0.01, ****P < 0.0001. Dotted lines denote epidermal boundaries. Scale bar: 200 μm

Notably, exogenous supplementation with PELI2 largely attenuated the inhibitory efficacy upon the depletion of ALKBH5, as demonstrated by Transwell (Fig. 6d and e) and wound scratch assays (Fig. 6f and g). To further characterize the gain-of-function of PELI2 in vivo, we overexpressed PELI2 using a lentiviral transfection vector (Lv-*PELI2*) in *Alkbh5*^‒/‒^ mice following previous lentiviral transfection strategy [32] (Fig. 7a). As expected, PELI2 was successfully overexpressed at the mRNA (Fig. 7b) and protein (Fig. 7c) levels in the epidermis of the wound edge of *Alkbh5*^‒/‒^ mice. Importantly, the restoration of PELI2 expression by lentiviral transfection significantly promoted the wound closure rate (Fig. 7d and e) and re-epithelialization capacity (Fig. 7f and g). Taken together, our data demonstrated that PELI2 functions as a necessary downstream effector of ALKBH5 in the regulation of cell migration and wound re-epithelialization.

### ALKBH5 stabilizes PELI2 mRNA in a YTHDF2-dependent manner

**Fig. 8:**
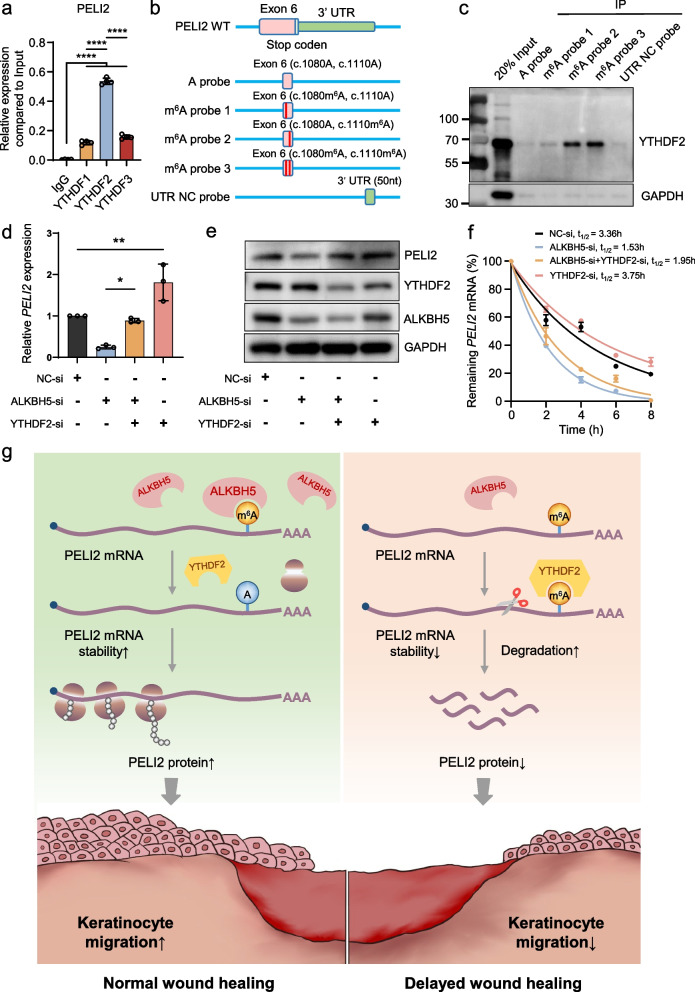
YTHDF2 binds to the m^6^A modification and stabilizes PELI2 mRNA. **a** RIP‒qPCR analysis revealed the enrichment of YTHDF1, YTHDF2, and YTHDF3 in the PELI2 transcript. The experiments were performed in quadruplicate, and the relative mRNA expression was compared to the input and is shown as the mean ± SD. One-way ANOVA, ****P < 0.0001. **b** Diagram showing the RNA probes used for RNA pull–down assays. **c** RNA pull–down of the endogenous YTHDF2 protein from cell extracts using PELI2 RNA probes with or without m^6^A modifications. Images are representative of three independent experiments. The full-length blots are presented in Additional file 8: Fig. S1. **d, e** PELI2 expression in ALKBH5- and YTHDF2-knockdown or control HaCaT cells was assessed by qRT‒PCR (**d**) and WB (**e**). The experiments were performed in triplicate. The relative mRNA expression level of PELI2 was quantified and is shown as the mean ± SD. One-way ANOVA, *P < 0.05, **P < 0.01. The full-length blots are presented in Additional file 8: Fig. S1. **f** ALKBH5- and YTHDF2-knockdown or control HaCaT cells were treated with actinomycin D (5 μg/mL) for 0, 2, 4, 6, and 8 h. The mRNA expression of PELI2 was analyzed by qRT–PCR. The experiments were performed in triplicate. **g** Schematic diagram showing how the reduction in m^6^A methylation mediated by the upregulation of the m^6^A eraser protein ALKBH5 facilitates wound re-epithelialization by enhancing PELI2 mRNA stability in a YTHDF2-dependent manner

Since YTHDF family members are the major component recognizing m^6^A-modified transcripts [6], we then attempted to identify the reader protein of methylated PELI2 mRNA. First, through RNA immunoprecipitation, we found that PELI2 presented with an abundant interacting frequency with YTHDF2; however, very limited signals were captured in the anti-YTHDF1/YTHDF3 groups (Fig. 8a). These data are in perfect alignment with the previous observation that YTHDF2 accelerates mRNA degradation of m^6^A-modified transcripts, which explains the mechanism underlying the inhibitory function of m^6^A in the regulation of PELI2 expression.

Subsequently, we analyzed the MeRIP-seq data and identified two RRACH motifs as potential m^6^A modification site in the last exon of PELI2 mRNA. Furthermore, to investigate the predominant m^6^A sites of PELI2, we designed biotin-labeled single-stranded RNA probes containing each (c.1080‒m^6^A, probe 1 and c.1110‒m^6^A, probe 2) or both m^6^A sites (probe 3) (Fig. 8b, Additional file 6: Table S6). Importantly, YTHDF2 specifically interacts with PELI2 probes containing the c.1110‒m^6^A-methylated site (probe 2 and probe 3), while other groups only showed negligible signals (Fig. 8c). This RNA pull‒down assay indicated that c.1110‒m^6^A methylation was recognized by YTHDF2, which agrees with the frequent binding between PELI2 mRNA and YTHDF2. Consistently, the silencing of YTHDF2 promoted PELI2 expression and rescued the ALKBH5-mediated downregulation of PELI2 expression at both the mRNA (Fig. 8d) and protein (Fig. 8e) levels, suggesting that YTHDF2 is the reader protein of methylated PELI2 mRNA.

Since YTHDF2 is responsible for RNA degradation [7], we then investigated whether the stability of PELI2 mRNA was regulated by YTHDF2. Intriguingly, knockdown of ALKBH5 resulted in the enhanced RNA stability of PELI2; however, this observation was attenuated by subsequent depletion of YTHDF2 (Fig. 8f). Together, our results provide new insight into the role of YTHDF2 in maintaining the stability of PELI2 mRNA by binding to its m^6^A modification site.

## Discussion

Despite the increased understanding of the role played by m^6^A modifications in controlling skin biology and disease pathogenesis [12, 14, 33], how m^6^A modification affects cutaneous wound re-epithelialization remains to be fully addressed. In the present study, we observed the specific upregulation of ALKBH5 expression in the epidermis at the wound edge, suggesting its unique role as a m^6^A regulator that is involved in the activation of skin repair. In vitro and in vivo studies showed that ALKBH5 facilitates keratinocyte migration as well as wound re-epithelialization. Moreover, the regulatory role of ALKBH5 in keratinocyte migration is mediated by removing the m^6^A modification from PELI2 mRNA and the subsequent enhancement of its stability in a YTHDF2-dependent manner. Taken together, these results highlight the important role of the m^6^A demethylase ALKBH5 in regulating keratinocyte function and wound re-epithelialization (Fig. 8g).

To our knowledge, we are the first to perform comprehensive m^6^A-MeRIP-seq in human epidermis and keratinocytes, which benefits a genome-wide understanding of m^6^A biology in the human epidermal barrier. These results showed an extensive involvement of m^6^A modifications in regulating keratinocyte differentiation, epidermal development, and wound healing processes. This evidence is also consistent with previous studies on mice, in which the highly m^6^A-modified genes in epidermal progenitors were found to be enriched in signaling pathways that regulate skin morphogenesis and hair follicle and epidermis renewal, such as the WNT, SHH, and NOTCH signaling pathways [14]. Defective hair follicle morphogenesis and maturation were observed in transgenic mice with conditional depletion of *METTL3* from K14-expressing keratinocytes, providing an example of epidermal and skin appendage development that is controlled by m^6^A modification [14]. In this study, we demonstrated that the global m^6^A modification was decreased in the wound edge skin using dot-blotting. After mapping of the expression of m^6^A “writers and erasers” at the wound edge, we further proposed that this decreased m^6^A level may be attributed to the upregulation of m^6^A demethylase ALKBH5 and FTO in both keratinocytes and fibroblasts at the wound edge. Our findings provide a direct evidence on the participation of m^6^A modification in regulating cutaneous wound healing.

Notably, our data provide the first evidence that the m^6^A demethylase ALKBH5 can promote the migration of keratinocytes. To date, the regulatory role of ALKBH5 in cell migration has been extensively investigated in different cell types, but these studies yielded inconsistent results. Some studies reported that ALKBH5 positively regulates the migration of trophoblast, uveal melanoma, and glioblastoma cell lines [34–36], while others observed opposite regulatory results in bladder cancer cells [37], cardiac microvascular endothelial cells [38], and colorectal cancer cells [39]. The exact reasons for these contradictory results are unclear; however, they may suggest a tissue- or cell-type-specific regulatory effects of ALKBH5, following a context-dependent manner. Here, we introduced a novel regulatory mechanism by which ALKBH5 modulates migration and re-epithelialization by removing m^6^A modifications in keratinocytes at the wound edge. Interestingly, ALKBH5 is also hyper-expressed in the dermis during wound healing, indicating that ALKBH5 may also play an important role in the dermal reconstruction (e.g., fibroblast activation). However, the underlying mechanism awaits successive studies.

FTO is another m^6^A demethylase with a similar function to ALKBH5 that is also upregulated in the wound edge epidermis. However, our results further showed that ALKBH5 could not regulate the expression of FTO and there is negligible compensatory expression of FTO upon ALKBH5 knockdown. As a result, we proposed that the upregulation of FTO in the wound edge epidermis is primary rather than secondary to ALKBH5 upregulation. Nevertheless, the regulatory role of FTO activation in the wound edge remain to be determined by future studies.

Notably, PELI2 is an E3-RING ubiquitin ligase that can catalyze the covalent attachment of ubiquitin moieties to substrate proteins [40]. The positive correlation of ALKBH5 and PELI2 expression has already been previously reported in endometrial cancer cells [41]. Here, we further identified a m^6^A-associated machinery through which ALKBH5 maintained PELI2 mRNA stability in a YTHDF2-dependent manner. In addition, our data highlighted that PELI2 accelerates keratinocyte migration and facilitate re-epithelialization during skin repair. Interestingly, keratinocytes expressing high levels of PELI2 colocalized with actively migrating keratinocytes according to the “leapfrog” model of re-epithelialization, which suggests that re-epithelialization is accomplished by suprabasal keratinocytes at the wound edge as they undergo morphological changes, reduce their desmosomal anchor levels, and tumble over basal keratinocytes [1, 42]. The highly specified localization of PELI2 in suprabasal keratinocytes at the wound edge also suggests a selective regulation of the behaviors of suprabasal keratinocytes. Although we have addressed that PELI2 plays an important role in keratinocyte activation during wound healing, the biological functions of PELI2 and its substrate in keratinocytes remains unknown. Previous studies and IPA analysis suggested roles for several potential mediators, including inflammatory cytokines (CXCL2, CXCL8, IL-6, and IL-1), ERK, TNF, and JNK signaling [43–48]. The detailed mechanism by which PELI2 regulates keratinocyte function in the context of skin repair warrants future investigation.

## Conclusion

In summary, this study revealed a novel mechanism underlying wound re-epithelialization by which the m^6^A eraser ALKBH5 is specifically activated at the wound edge and facilitates keratinocyte migration by removing the m^6^A sites from PELI2 mRNA and protecting it from YTHDF2-mediated degradation. This study extends our understanding of wound re-epithelialization from the perspective of RNA epitranscriptomic regulation. Importantly, ALKBH5 and PELI2 could be potential therapeutic targets in the development of a reprogrammed m^6^A targeted therapy for patients with refractory wounds.

## Supplementary Information

**Additional file 1: Table S1.** Clinical information of patients with chronic wounds.

**Additional file 2: Table S2.** Oligonucleotides used for siRNA expression vector.

**Additional file 3: Table S3.** Primers used for PELI2 overexpression plasmid.

**Additional file 4: Table S4.** Antibodies used in experiments.

**Additional file 5: Table S5.** Primers used in experiments.

**Additional file 6: Table S6.** ssRNA probes used for RNA pull‒down assay.

**Additional file 7: Table S7.** Donor’s information of human epidermis tissue.

**Additional file 8: Fig. S1.** Uncropped original western blots.

**Additional file 9: Fig. S2.** The expression of ALKBH5 in the epidermis and dermis of normal skin and wound edge. Dotted lines denote epidermal boundaries. Epi, epidermis. Scale bar: 100 μm.

**Additional file 10: Fig. S3.** The expression of METTL3, METTL14, FTO, and ALKBH5 in keratinocytes and fibroblasts at the normal skin and wound edge. Dotted lines denote epidermal boundaries. Epi, epidermis. Scale bar: 100 μm.

**Additional file 11: Fig. S4.** The expression of FTO upon ALKBH5 depletion in vitro and in vivo*.* a. The expression of FTO mRNA was measured in HaCaT cells after ALKBH5 knockdown using RNA‒seq and qRT‒PCR. RNA‒seq was performed in duplicate and was deposited in the GEO database. qRT‒PCR was performed in triplicate, and the relative mRNA expression is shown as mean ± SD. One‒way ANOVA, ns, not significant. b. The expression of FTO protein was measured in HaCaT cells after ALKBH5 knockdown using WB. The full-length blots are presented in Additional file 8: Fig. S1. c. Statistical analysis of WB. Experiments were performed in triplicate. The relative expression of FTO is shown as mean ± SD. One‒way ANOVA, ns, not significant. d. The expression of FTO mRNA was measured in the skin of *Alkbh5*^*‒/‒*^ and WT mice using qRT‒PCR. Three animals in each group were included for analysis. Relative mRNA expression is shown as mean ± SD. T test, not significant. e. The expression of FTO protein was measured in the skin of *Alkbh5*^*‒/‒*^ and WT mice using WB. The full-length blots are presented in Additional file 8: Fig. S1. f. Statistical analysis of WB. Three animals in each group were included for analysis. The relative expression of FTO is shown as mean ± SD. T test, not significant.

**Additional file 12: Fig. S5.** Inhibition of ALKBH5 showed no effects on keratinocyte proliferation and apoptosis in vitro*.* a. Proliferation of ALKBH5‒knockdown or control HaCaT cells after siRNA transfection was analyzed by CCK‒8 assay. The experiments were performed in triplicate. One‒way ANOVA, ns, not significant. b, c. Proliferation of ALKBH5‒knockdown or control HaCaT cells was analyzed by Ed‒U staining assay. All of the experiments were performed in triplicate, and five random fields were included in the analysis. One‒way ANOVA, ns, not significant. Scale bar: 100 μm. d, e. Cell cycle of ALKBH5‒knockdown or control HaCaT cells was analyzed by flow cytometry. All of the experiments were performed in triplicate. One‒way ANOVA revealed no significant difference. f, g. Apoptosis of ALKBH5‒knockdown or control HaCaT cells was analyzed by flow cytometry. All of the experiments were performed in triplicate. One‒way ANOVA revealed no significant difference.

**Additional file 13: Fig. S6.** H&E staining of normal skin from WT and *Alkbh5*^*‒/‒*^ mice. Scale bar: 100 μm.

**Additional file 14: Fig. S7.** The proliferation of epidermal cells of WT and *Alkbh5*^‒/‒^ mice at PWD8. a. IF showed representative Ki67^+^ cell distribution in the wound edge of WT and *Alkbh5*^‒/‒^ mice at PWD8. Dotted lines denote epidermal boundaries. Epi, epidermis. Scale bar: 100 μm. b. Statistical analysis of the percentage of Ki67^+^ proliferating keratinocytes per HPF. Twelve wound samples were collected from six mice, and the percentage of Ki67^+^ cells is shown as the mean ± SD. T test, ns, not significant.

**Additional file 15: Table S8**. The total m^6^A peaks identified by MeRIP‒seq.

**Additional file 16: Fig. S8.** Genome‒wide mapping of m^6^A modification in human epidermis and epidermal cell line. a. The distribution of m^6^A sites along the length of mRNA transcripts. b. The stacked bar chart showing the m^6^A peak distribution in different RNA regionsin human epidermis and keratinocyte cell lines. c. Top enriched motifs within m^6^A peaks that were identified in the human epidermis and keratinocyte cell lines. d. The number of genes with m^6^A peaks in the 3’UTR of mRNA transcripts. e. Venn diagram showing 3710 common genes with m^6^A peaks in the 3’UTRs from four different samples.

**Additional file 17: Fig. S9.** GO enrichment map showing the molecular functions of 3710 genes with m^6^A peaks in the 3’UTRs of mRNA transcripts.

**Additional file 18: Fig. S10.** Gene ontology analysis and a Circus plot of 16 potential target genes of ALKBH5. These genes were associated with the regulation of epithelium development; cellular functions such as epithelial cell differentiation, cell division, cell migration, and cytoskeleton organization; and signaling pathways including Wnt signaling

**Additional file 19: Fig. S11.** The correlation of PELI2 with cell migration as indicated by IPA analysis. a. The molecular regulatory network by which PELI2 functions in cell migration as predicted by IPA. b. IPA predicted the regulatory network by which PELI2 functions in the migration of keratinocytes or epidermal cells and the wound healing process.

**Additional file 20: Fig. S12.** The expression of PELI2 in keratinocytes and fibroblasts at the normal skin and wound edge. Dotted lines denote epidermal boundaries. Epi, epidermis. Scale bar: 100 μm.

**Additional file 21: Fig. S13.** The characterization of primary keratinocytes by IF staining of specific markers. IF showing the expression of the keratinocyte marker keratin 14 and the fibroblast marker fibronectin 1in primary keratinocytes and fibroblasts extracted from the skin of WT and *Alkbh5*^‒/‒^mice. A keratinocyte cell lineand fibroblast cell linewere used as references. Scale bar: 50 μm. KC, keratinocyte; Fb, fibroblast.

## Data Availability

MeRIP-seq and RNA-seq data have been deposited in the NCBI Gene Expression Omnibus (GEO), under accession codes GSE 211442 and GSE 211076, respectively. All other data supporting the key findings of this study are available within the article and supplementary information files or from the corresponding author upon reasonable request.

## Abbreviations

m^6^A: *N*^6^-Methyladenosine RNA modification
ALKBH5: α-Ketoglutarate-dependent dioxygenase alkB homolog 5
PELI2: Pellino E3 ubiquitin protein ligase family member 2
YTHDF2: YTH *N*^6^-methyladenosine RNA binding protein 2
METTL3: Methyltransferase-like 3
METTL14: Methyltransferase-like 14
WTAP: Wilms tumor 1-associated protein
FTO: Fat‒mass and obesity-associated protein
HNRNP: Heterogeneous nuclear ribonucleoprotein
IGF2BP: Insulin-like growth factor 2 mRNA-binding proteins
MeRIP-seq: Methylated RNA immunoprecipitation sequencing
WB: Western blotting
DB: Dot-blotting
IF: Immunofluorescence
siRNAs: Small interfering RNAs
ssRNA: Single-stranded RNA
KO: Knockout
WT: Wild type
IPA: Ingenuity pathway analysis
qRT‒PCR: Quantitative real-time polymerase chain reaction
mRNA: Messenger RNA
Lv: Lentiviral
K14: Keratin 14
RIP: RNA immunoprecipitation
PWD: Post wound day
HaCaT: Human immortal keratinocyte line
STR: Short tandem repeat
RRACH: R = G or A; H = A, C or U
A, C, G, U: Stands for adenine, cytosine, guanine and uracil respectively
