# Supplementary material for: ALKBH5-mediated m6A demethylation fuels cutaneous wound re-epithelialization by enhancing PELI2 mRNA stability
Source: Inflamm Regen. 2023 Jul 14;43:36. doi: 10.1186/s41232-023-00288-0 (PMC10347733; doi:10.1186/s41232-023-00288-0)
Supplement: Supplementary file 1 — Additional file 1: Table S1. Clinical information of patients with chronic wounds. [file 41232_2023_288_MOESM1_ESM.docx]

**Table S1. Clinical information of patients with chronic wounds**

| Order | Sex | Age | Causes | Location | Ulceration | Infection | Comorbidities |
| --- | --- | --- | --- | --- | --- | --- | --- |
| 1 | Male | 45 | Diabetic wounds | Buttock | + | ‒ | CVD |
| 2 | Female | 56 | Diabetic wounds | Lower extremity | + | ‒ | ‒ |
| 3 | Male | 68 | Diabetic wounds | Hand | + | ‒ | ‒ |
| 4 | Male | 73 | Diabetic wounds | Foot | ‒ | ‒ | Anemia |
| 5 | Male | 63 | Diabetic wounds | Foot | + | ‒ | ‒ |
| 6 | Female | 53 | Diabetic wounds | Buttock | ‒ | ‒ | CVD |
| 7 | Female | 48 | Diabetic wounds | Foot | + | ‒ | ‒ |
| 8 | Male | 72 | Diabetic wounds | Neck | ‒ | ‒ | ‒ |
| 9 | Male | 66 | Diabetic wounds | Abdomen | ‒ | ‒ | ‒ |
| 10 | Male | 56 | Diabetic wounds | Upper  extremity | + | ‒ | Renal insufficiency |

CVD, cardiovascular disease.
