# Supplementary material for: ALKBH5-mediated m6A demethylation fuels cutaneous wound re-epithelialization by enhancing PELI2 mRNA stability
Source: Inflamm Regen. 2023 Jul 14;43:36. doi: 10.1186/s41232-023-00288-0 (PMC10347733; doi:10.1186/s41232-023-00288-0)
Supplement: Supplementary file 2 — Additional file 2: Table S2. Oligonucleotides used for siRNA expression vector. [file 41232_2023_288_MOESM2_ESM.docx]

**Table S2. Oligonucleotides used for siRNA expression vector**

| Oligonucleotides name | Sequence (5’‒3’) |
| --- | --- |
| ALKBH5‒si1‒sense | ACAAGUACUUCUUCGGCGAdTdT |
| ALKBH5‒si1‒antisense | UCGCCGAAGAAGUACUUGUdTdT |
| ALKBH5‒si2‒sense | GCGCCGUCAUCAACGACUAdTdT |
| ALKBH5‒si2‒antisense | UAGUCGUUGAUGACGGCGCdTdT |
| YTHDF2‒si‒sense | CCUAGGUAGCACUCCAUUUCUTT |
| YTHDF2‒si‒antisense | AGAAAUGGAGUGCUACCUAGGTT |
