# Supplementary material for: ALKBH5-mediated m6A demethylation fuels cutaneous wound re-epithelialization by enhancing PELI2 mRNA stability
Source: Inflamm Regen. 2023 Jul 14;43:36. doi: 10.1186/s41232-023-00288-0 (PMC10347733; doi:10.1186/s41232-023-00288-0)
Supplement: Supplementary file 3 — Additional file 3: Table S3. Primers used for PELI2 overexpression plasmid. [file 41232_2023_288_MOESM3_ESM.docx]

**Table S3. Primers used for PELI2 overexpression plasmid (****GTP‒C‒3Flag‒H‒PELI2‒WT)**

| Oligonucleotides name | Sequence (5’‒3’) |
| --- | --- |
| PELI2‒forwad | TAGAGCTAGCGAATTCATGTTTTCCCCTGGCCAG |
| PELI2‒reverse | CTTTGTAGTCGGATCCGTCAATTGGACCTTGGAAAATTAA |
