# Supplementary material for: ALKBH5-mediated m6A demethylation fuels cutaneous wound re-epithelialization by enhancing PELI2 mRNA stability
Source: Inflamm Regen. 2023 Jul 14;43:36. doi: 10.1186/s41232-023-00288-0 (PMC10347733; doi:10.1186/s41232-023-00288-0)
Supplement: Supplementary file 4 — Additional file 4: Table S4. Antibodies used in experiments. [file 41232_2023_288_MOESM4_ESM.docx]

**Table S4. Antibodies used in experiments**

| Antibodies | Source | Identifier | Dilution |
| --- | --- | --- | --- |
| anti‒ALKBH5 | Abcam | ab195377 | IHC‒P: 1:400; WB: 1:1000 |
| anti‒PELI2 | Proteintech | Cat No. 16097‒1‒AP | IHC‒P: 1:400; WB: 1:1000 |
| anti‒FTO | Proteintech | Cat No. 27226‒1‒AP | IHC‒P: 1:400; WB: 1:1000 |
| anti‒METTL3 | Proteintech | Cat No. 15073‒1‒AP | IHC‒P: 1:400 |
| anti‒METTL14 | Proteintech | Cat No. 26158‒1‒AP | IHC‒P: 1:400 |
| anti‒Keratin 14 | CST | # 48020S | IHC‒P: 1:500 |
| anti‒Ki67 | CST | # 9129S | IHC‒P: 1:400 |
| anti-PDGFRα | Abcam | ab96569 | IHC‒P: 1:400 |
| anti‒Flag | Millipore | F7425 | WB: 1:5000 |
| anti‒m^6^A | Abclonal | A19841 | RIP: 5μg; DB: 1:1000 |
| anti‒GAPDH | CST | # 5174S | WB: 1:2000 |
| anti‒YTHDF1 | Proteintech | Cat No. 17479‒1‒AP | RIP: 5μg; WB: 1:1000 |
| anti‒YTHDF2 | Proteintech | Cat No. 24744‒1‒AP | RIP: 5μg; WB: 1:1000 |
| anti‒YTHDF3 | Proteintech | Cat No. 25537‒1‒AP | RIP: 5μg; WB: 1:1000 |
| Normal rabbit IgG antibody | Millipore | Cat#PP64 | RIP: 5μg |
| Anti‒rabbit IgG, HRP‒linked Antibody | CST | #7074S | WB: 1:2500 |
| Anti‒mouse IgG (H+L), F(ab')2 Fragment (Alexa Fluor® 488 Conjugate) | CST | #4408S | IHC‒P: 1:400 |
| Anti‒rabbit IgG (H+L), F(ab')2 Fragment (Alexa Fluor® 594 Conjugate) | CST | #8889S | IHC‒P: 1:400 |
| Anti‒rabbit IgG (H+L), F(ab')2 Fragment (Alexa Fluor® 488 Conjugate) | CST | #4412 | IHC‒P: 1:400 |
| Anti‒mouse IgG (H+L), F(ab')2 Fragment (Alexa Fluor® 594 Conjugate) | CST | #8890 | IHC‒P: 1:400 |
