# Supplementary material for: ALKBH5-mediated m6A demethylation fuels cutaneous wound re-epithelialization by enhancing PELI2 mRNA stability
Source: Inflamm Regen. 2023 Jul 14;43:36. doi: 10.1186/s41232-023-00288-0 (PMC10347733; doi:10.1186/s41232-023-00288-0)
Supplement: Supplementary file 5 — Additional file 5: Table S5. Primers used in experiments. [file 41232_2023_288_MOESM5_ESM.docx]

**Table S5. Primers used in experiments**

| Primer sequences for qRT‒PCR | | | |
| --- | --- | --- | --- |
| ALKBH5 | Human | Forward | 5’‒ATCCTCAGGAAGACAAGATTAG‒3’ |
|  |  | Reverse | 5’‒TTCTCTTCCTTGTCCATCTC‒3’ |
| FTO | Human | Forward | 5’‒TGGATGAGCCAGCTTCACTG‒3’ |
|  |  | Reverse | 5’‒CCCTGTTGCACATTCCCTGA‒3’ |
| PELI2 | Human | Forward | 5’‒CAGCTGCAAAGGTCAACACA‒3’ |
|  |  | Reverse | 5’‒GTGTGTACTCCACCACCACA‒3’ |
| C3orf33 | Human | Forward | 5’‒GCCTAGTCCGGAACATCAGC‒3’ |
|  |  | Reverse | 5’‒GGCGTAATCGTCCACGTAGT‒3’ |
| CDK5R1 | Human | Forward | 5’‒CTCAGTGTGAAGCCTGTCGT‒3’ |
|  |  | Reverse | 5’‒GCTTGTTCTGTGCATGGGTG‒3’ |
| BAMBI | Human | Forward | 5’‒CCACTCTGGCACCACCATAC‒3’ |
|  |  | Reverse | 5’‒ATCGTGCAGCCCTCTGTAAT‒3’ |
| PGBD2 | Human | Forward | 5’‒TGGGGTAGGGCAGGTTCTTA‒3’ |
|  |  | Reverse | 5’‒AGGGTGCATGCTTCTGGAAA‒3’ |
| PLEKHO1 | Human | Forward | 5’‒CTTCGAAGCCGCCTTCTTCC‒3’ |
|  |  | Reverse | 5’‒TCAGCACCACATAGCGGTTT‒3’ |
| RHOF | Human | Forward | 5’‒GAGCTGAAGATCGTGATCGTGG‒3’ |
|  |  | Reverse | 5’‒AGCCGGTCATAGTCTTCTTGC‒3’ |
| TP53I3 | Human | Forward | 5’‒GGCACAGCTGCTATCCAACT‒3’ |
|  |  | Reverse | 5’‒AGCATTTGCTTGTACTTTGGTGA‒3’ |
| ZNF20 | Human | Forward | 5’‒CCAGAAGCCGGGAAATGGAT‒3’ |
|  |  | Reverse | 5’‒AGAGATGAATGACCCGTGCC‒3’ |
| ZNF573 | Human | Forward | 5’‒ACATGGCCAAAGTCACAAGGA‒3’ |
|  |  | Reverse | 5’‒CAGGGCTCTTTTCCTCGCTC‒3’ |
| ZNF577 | Human | Forward | 5’‒GGTCATCGGTCTGTTGAGCA‒3’ |
|  |  | Reverse | 5’‒CCTTCCCAGAAGTGGTGGTC‒3’ |
| ZNF785 | Human | Forward | 5’‒AGGAAAGGAAGTGGGCGTTT‒3’ |
|  |  | Reverse | 5’‒GGCACTGGAATGTAGGGGAC‒3’ |
| ZBED6 | Human | Forward | 5’‒CACCTCCAGTTCAGGTTCTGT‒3’ |
|  |  | Reverse | 5’‒ACAACTGCTACAGCAGAAGGG‒3’ |
| UCN | Human | Forward | 5’‒TACAGCTGTGCCCTGGGA‒3’ |
|  |  | Reverse | 5’‒CCAGCAGCAAGAGGAGCG‒3’ |
| FOXJ1 | Human | Forward | 5’‒CACGTGAAGCCTCCCTACTC‒3’ |
|  |  | Reverse | 5’‒CCACTTGTAGATGGCCGACA‒3’ |
| FZD8 | Human | Forward | 5’‒GGAGTGGGGTTACCTGTTGG‒3’ |
|  |  | Reverse | 5’‒GTAGCCGATGCCCTTACACA‒3’ |
| GAPDH | Human | Forward | 5’‒ ATGTTGCAACCGGGAAGGA‒3’ |
|  |  | Reverse | 5’‒ CAGGAGCGCAGGGTTAGTC‒3’ |
| ALKBH5 | Mouse | Forward | 5’‒ACTGTGCTCAGTGGGTATGC‒3’ |
|  |  | Reverse | 5’‒TTCCAATCGCGGTGCATCTA‒3’ |
| PELI2 | Mouse | Forward | 5’‒TACGGATAGCTCCGGGGAAG‒3’ |
|  |  | Reverse | 5’‒ACACTCCCATCCCGAAAACG‒3’ |
| FTO | Mouse | Forward | 5’‒TTGGGACATCGAGACACCAG‒3’ |
|  |  | Reverse | 5’‒ATTTCAACGAGACGTCGCCA‒3’ |
| METTL3 | Mouse | Forward | 5’‒GGACTCTGGGCACTTGGATTTA‒3’ |
|  |  | Reverse | 5’‒CAGGTGCATCTGGCGTAGAG‒3’ |
| METTL14 | Mouse | Forward | 5’‒CTCCAGGTCGGAGTGTGAAC‒3’ |
|  |  | Reverse | 5’‒AACCGTTTAAACCAGCCCCT‒3’ |
| ALKBH5 | Mouse | Forward | 5’‒ACTGTGCTCAGTGGGTATGC‒3’ |
|  |  | Reverse | 5’‒TTCCAATCGCGGTGCATCTA‒3’ |
| WTAP | Mouse | Forward | 5’‒CATTTTGTGGCTGCGAGACC‒3’ |
|  |  | Reverse | 5’‒TCTGTTTCACTCAGTCGGACC‒3’ |
| GAPDH | Mouse | Forward | 5’‒CAGTGGCAAAGTGGAGATTGTTG‒3’ |
|  |  | Reverse | 5’‒TCGCTCCTGGAAGATGGTGAT‒3’ |
| Primer sequences for RNA Binding Protein Immunoprecipitation (RIP) | | | |
| PELI2 | Human | Forward | 5’‒ATCAGAGGAGGGTGACAGGG‒3’ |
|  |  | Reverse | 5’‒GACTTCAATGACGACGCTGC‒3’ |
