# Supplementary material for: ALKBH5-mediated m6A demethylation fuels cutaneous wound re-epithelialization by enhancing PELI2 mRNA stability
Source: Inflamm Regen. 2023 Jul 14;43:36. doi: 10.1186/s41232-023-00288-0 (PMC10347733; doi:10.1186/s41232-023-00288-0)
Supplement: Supplementary file 6 — Additional file 6: Table S6. ssRNA probes used for RNA pull‒down assay. [file 41232_2023_288_MOESM6_ESM.docx]

**Table S6. ssRNA probes used for RNA pull‒down assay**

| Oligonucleotides name | Sequence (5’‒3’) |
| --- | --- |
| A probe | TAGACGCAGGACCGCCAACTCATGCTTTCACTCCCTGTGGACACGTGTGC‒Biotin |
| m^6^A probe 1 | TAGACGCAGGm^6^ACCGCCAACTCATGCTTTCACTCCCTGTGGACACGTGTGC‒Biotin |
| m^6^A probe 2 | TAGACGCAGGACCGCCAACTCATGCTTTCACTCCCTGTGGm^6^ACACGTGTGC‒Biotin |
| m^6^A probe 3 | TAGACGCAGGm^6^ACCGCCAACTCATGCTTTCACTCCCTGTGGm^6^ACACGTGTGC‒Biotin |
| UTR NC probe | TTCTGAAAATCCCCTACATTTTTTAATTAAAGAAATTTCCTTGGTGCCTA‒Biotin |
