# Supplementary material for: ALKBH5-mediated m6A demethylation fuels cutaneous wound re-epithelialization by enhancing PELI2 mRNA stability
Source: Inflamm Regen. 2023 Jul 14;43:36. doi: 10.1186/s41232-023-00288-0 (PMC10347733; doi:10.1186/s41232-023-00288-0)
Supplement: Supplementary file 7 — Additional file 7: Table S7. Donor’s information of human epidermis tissue. [file 41232_2023_288_MOESM7_ESM.docx]

**Table S7. Donor’s information of human epidermis tissue**

| Sample name | Gender | Age | Location | Comorbidities |
| --- | --- | --- | --- | --- |
| Epi-Rep1 | Male | 23 | Foreskin | ‒ |
| Epi-Rep2 | Male | 34 | Foreskin | ‒ |
