# Supplementary figures and images for: ALKBH5-mediated m6A demethylation fuels cutaneous wound re-epithelialization by enhancing PELI2 mRNA stability
Source: Inflamm Regen. 2023 Jul 14;43:36. doi: 10.1186/s41232-023-00288-0 (PMC10347733; doi:10.1186/s41232-023-00288-0)

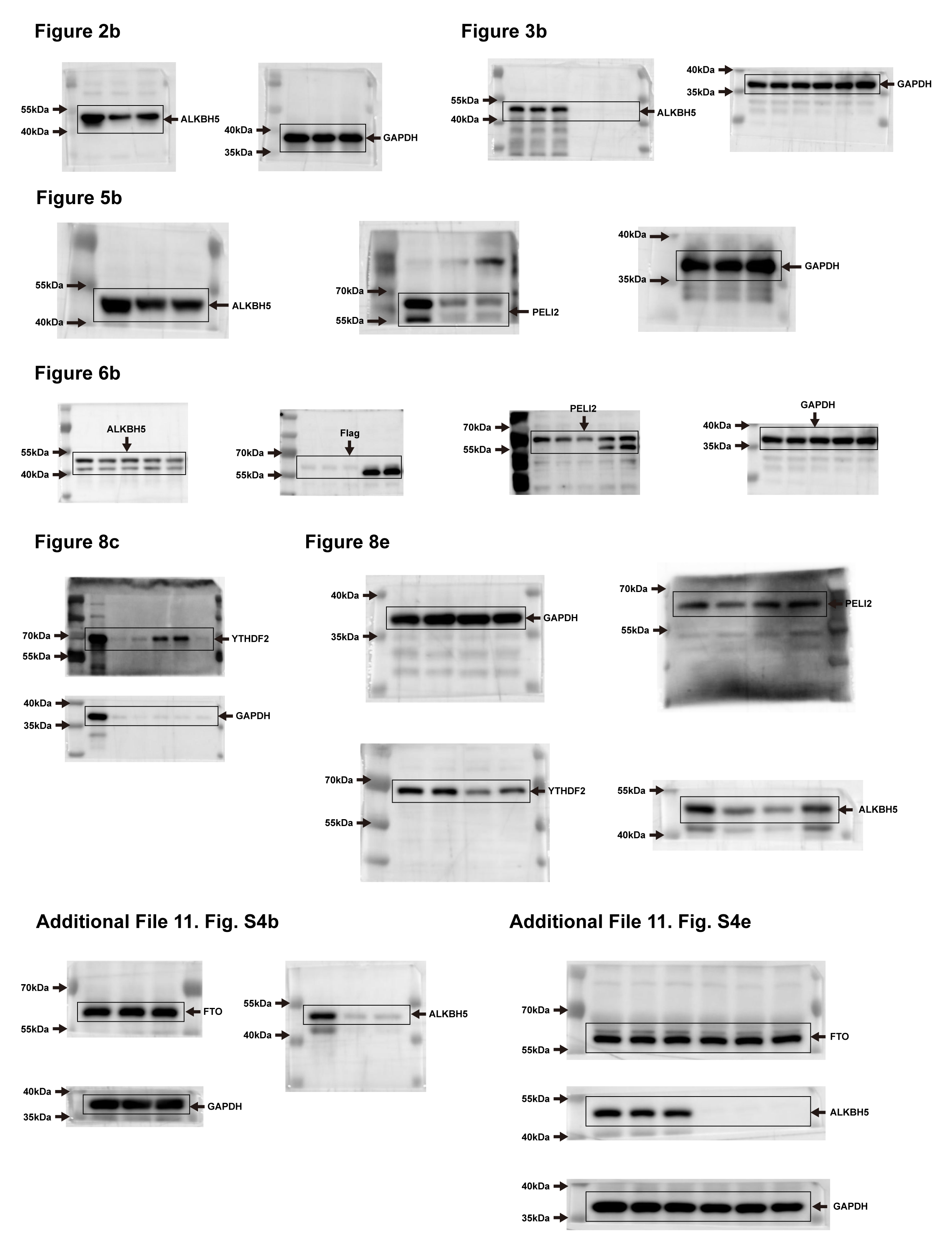

Supplement: Supplementary file 8 — Additional file 8: Fig. S1. Uncropped original western blots. [file 41232_2023_288_MOESM8_ESM.tif]

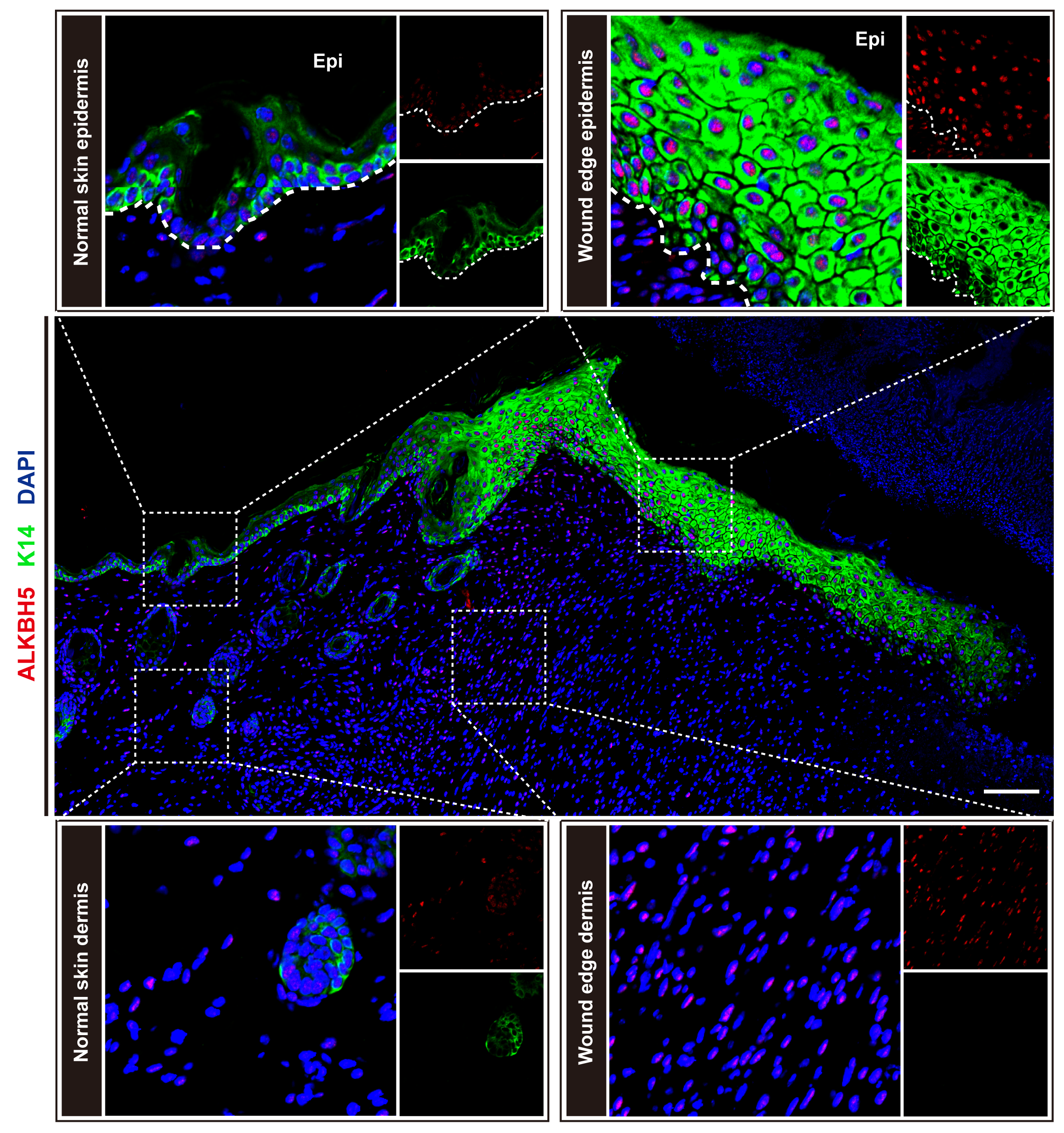

Supplement: Supplementary file 9 — Additional file 9: Fig. S2. The expression of ALKBH5 in the epidermis and dermis of normal skin and wound edge. Dotted lines denote epidermal boundaries. Epi, epidermis. Scale bar: 100 μm. [file 41232_2023_288_MOESM9_ESM.tif]

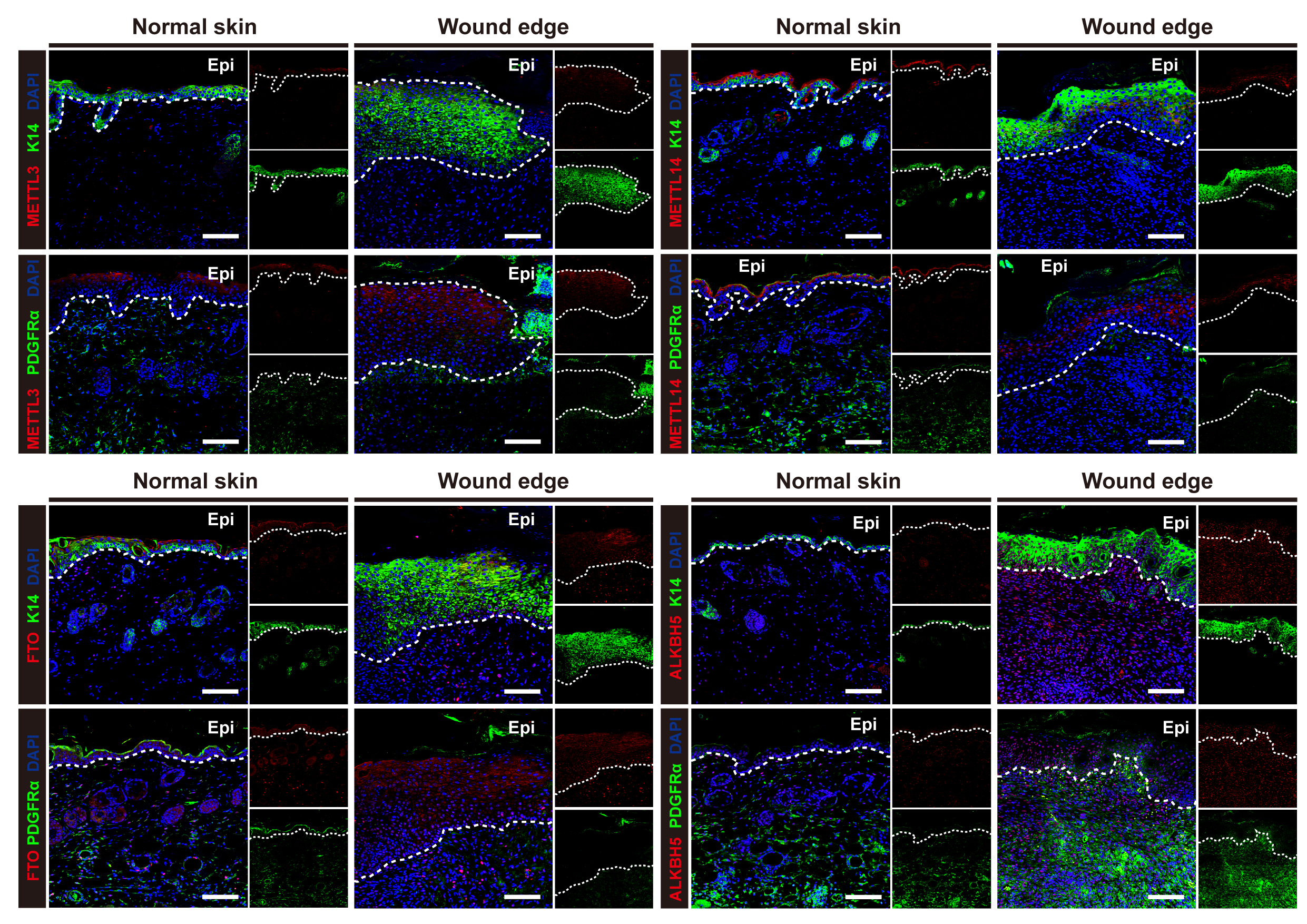

Supplement: Supplementary file 10 — Additional file 10: Fig. S3. The expression of METTL3, METTL14, FTO, and ALKBH5 in keratinocytes and fibroblasts at the normal skin and wound edge. Dotted lines denote epidermal boundaries. Epi, epidermis. Scale bar: 100 μm. [file 41232_2023_288_MOESM10_ESM.tif]

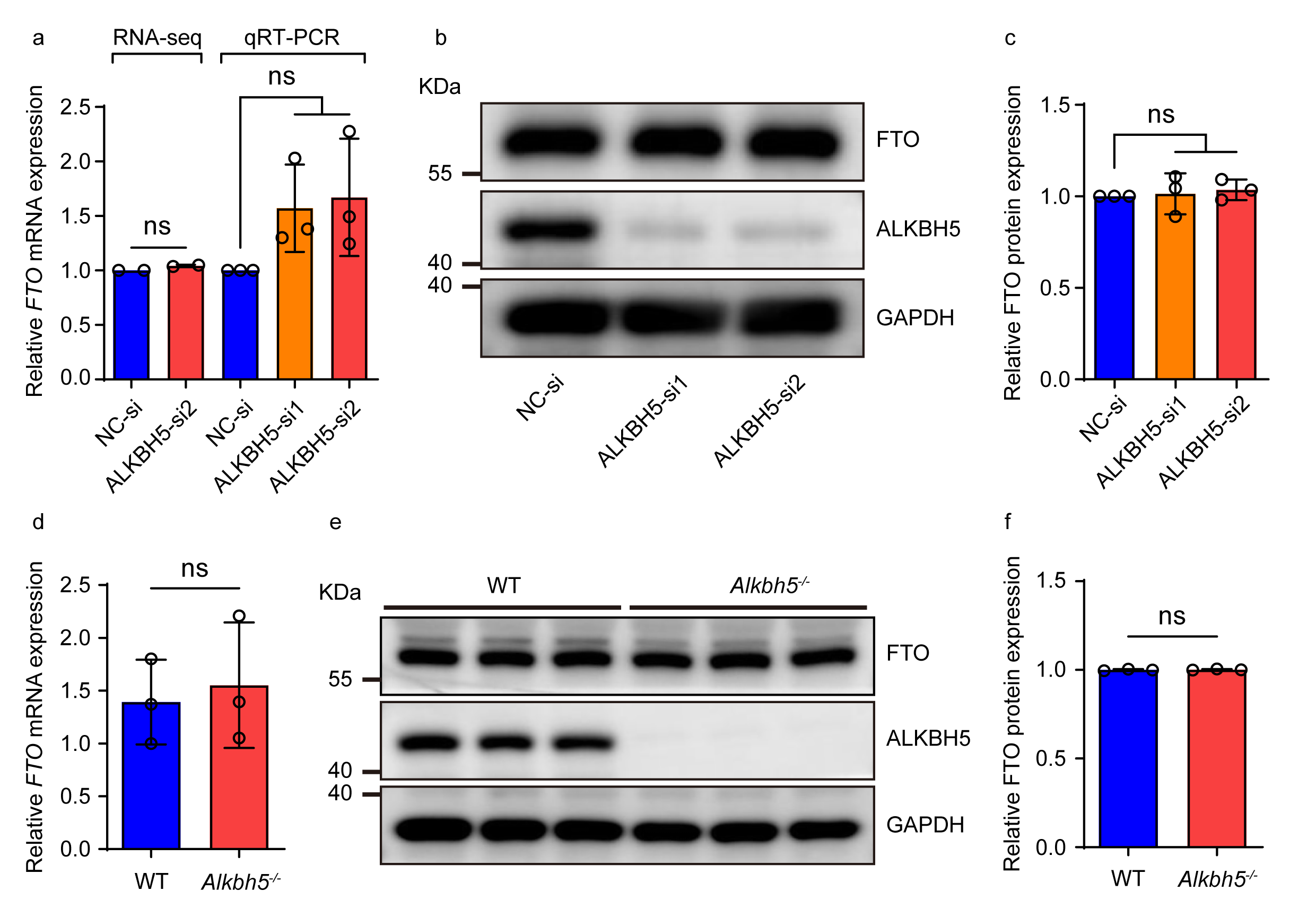

Supplement: Supplementary file 11 — Additional file 11: Fig. S4. The expression of FTO upon ALKBH5 depletion in vitro and in vivo. a. The expression of FTO mRNA was measured in HaCaT cells after ALKBH5 knockdown using RNA‒seq and qRT‒PCR. RNA‒seq was performed in duplicate and was deposited in the GEO database. qRT‒PCR was performed in triplicate, and the relative mRNA expression is shown as mean ± SD. One‒way ANOVA, ns, not significant. b. The expression of FTO protein was measured in HaCaT cells after ALKBH5 knockdown using WB. The full-length blots are presented in Additional file 8: Fig. S1. c. Statistical analysis of WB. Experiments were performed in triplicate. The relative expression of FTO is shown as mean ± SD. One‒way ANOVA, ns, not significant. d. The expression of FTO mRNA was measured in the skin of Alkbh5‒/‒ and WT mice using qRT‒PCR. Three animals in each group were included for analysis. Relative mRNA expression is shown as mean ± SD. T test, not significant. e. The expression of FTO protein was measured in the skin of Alkbh5‒/‒ and WT mice using WB. The full-length blots are presented in Additional file 8: Fig. S1. f. Statistical analysis of WB. Three animals in each group were included for analysis. The relative expression of FTO is shown as mean ± SD. T test, not significant. [file 41232_2023_288_MOESM11_ESM.tif]

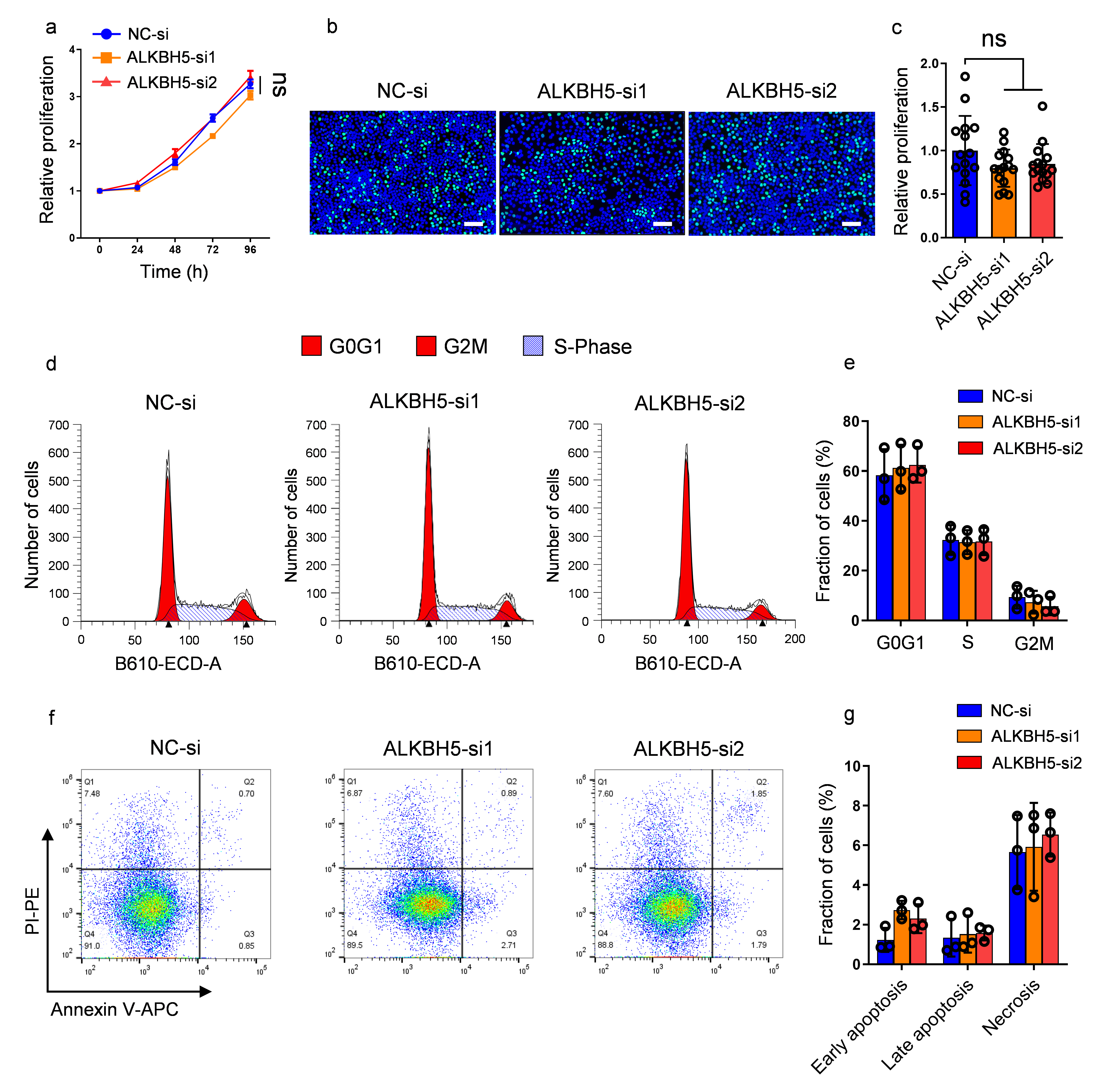

Supplement: Supplementary file 12 — Additional file 12: Fig. S5. Inhibition of ALKBH5 showed no effects on keratinocyte proliferation and apoptosis in vitro. a. Proliferation of ALKBH5‒knockdown or control HaCaT cells after siRNA transfection was analyzed by CCK‒8 assay. The experiments were performed in triplicate. One‒way ANOVA, ns, not significant. b, c. Proliferation of ALKBH5‒knockdown or control HaCaT cells was analyzed by Ed‒U staining assay. All of the experiments were performed in triplicate, and five random fields were included in the analysis. One‒way ANOVA, ns, not significant. Scale bar: 100 μm. d, e. Cell cycle of ALKBH5‒knockdown or control HaCaT cells was analyzed by flow cytometry. All of the experiments were performed in triplicate. One‒way ANOVA revealed no significant difference. f, g. Apoptosis of ALKBH5‒knockdown or control HaCaT cells was analyzed by flow cytometry. All of the experiments were performed in triplicate. One‒way ANOVA revealed no significant difference. [file 41232_2023_288_MOESM12_ESM.tif]

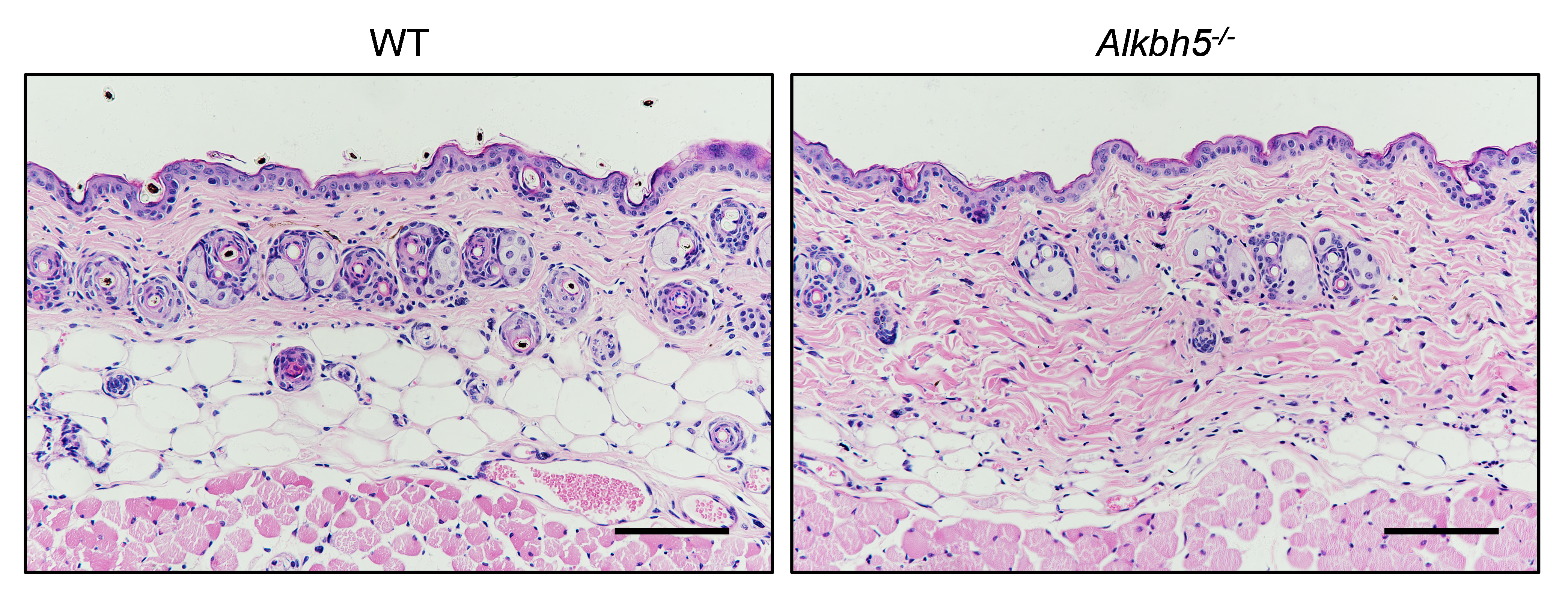

Supplement: Supplementary file 13 — Additional file 13: Fig. S6. H&E staining of normal skin from WT and Alkbh5‒/‒ mice. Scale bar: 100 μm. [file 41232_2023_288_MOESM13_ESM.tif]

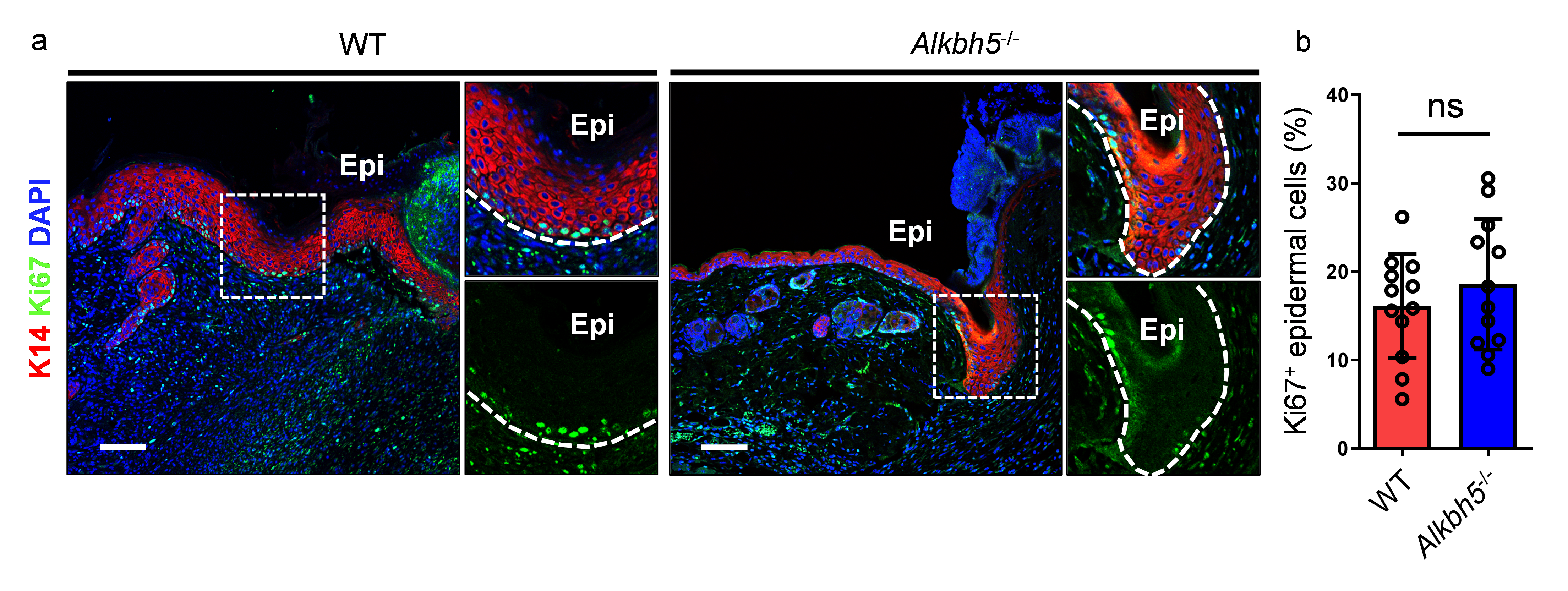

Supplement: Supplementary file 14 — Additional file 14: Fig. S7. The proliferation of epidermal cells of WT and Alkbh5‒/‒ mice at PWD8. a. IF showed representative Ki67+ cell distribution in the wound edge of WT and Alkbh5‒/‒ mice at PWD8. Dotted lines denote epidermal boundaries. Epi, epidermis. Scale bar: 100 μm. b. Statistical analysis of the percentage of Ki67+ proliferating keratinocytes per HPF. Twelve wound samples were collected from six mice, and the percentage of Ki67+ cells is shown as the mean ± SD. T test, ns, not significant. [file 41232_2023_288_MOESM14_ESM.tif]

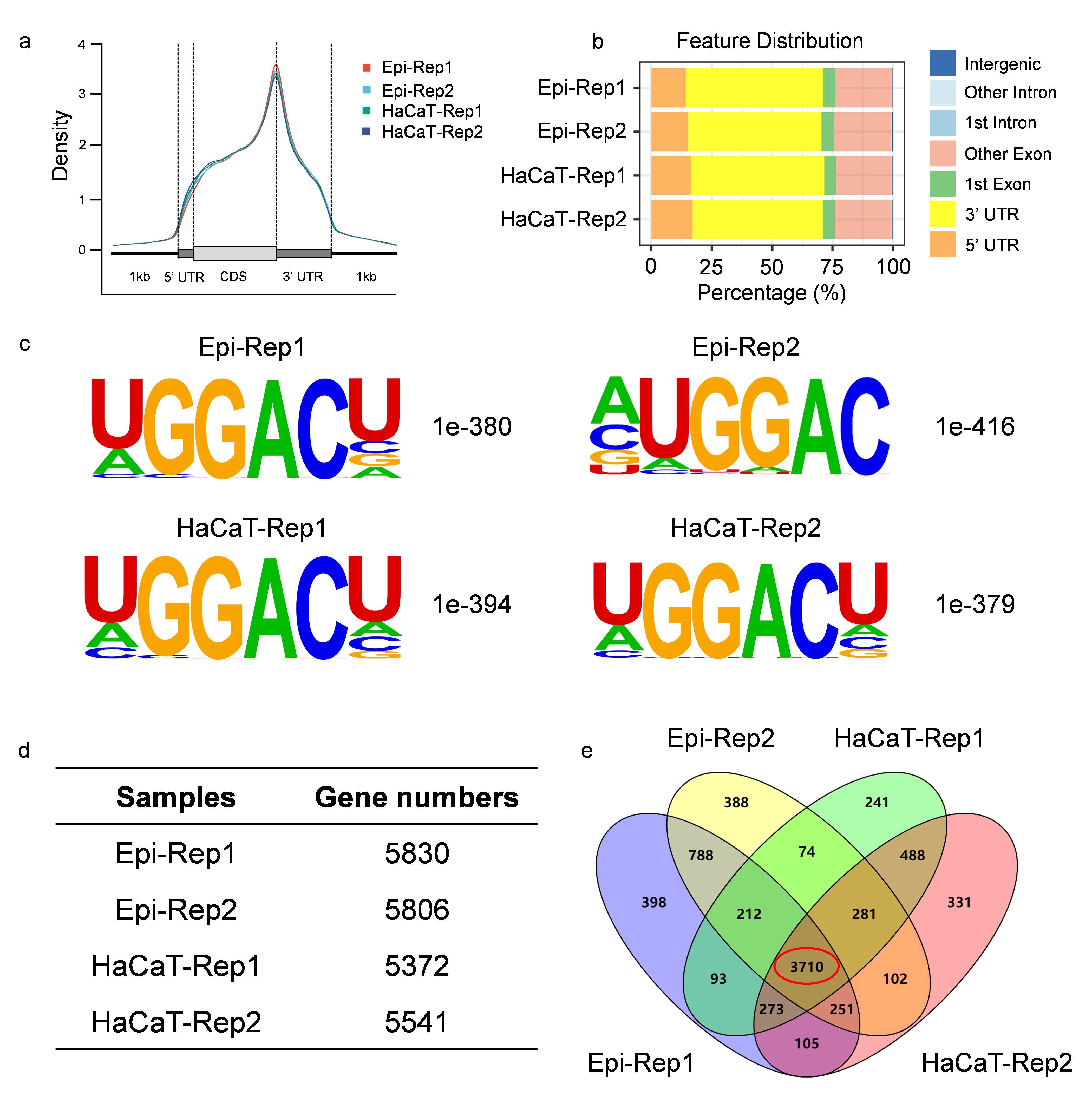

Supplement: Supplementary file 16 — Additional file 16: Fig. S8. Genome‒wide mapping of m6A modification in human epidermis and epidermal cell line. a. The distribution of m6A sites along the length of mRNA transcripts. b. The stacked bar chart showing the m6A peak distribution in different RNA regionsin human epidermis and keratinocyte cell lines. c. Top enriched motifs within m6A peaks that were identified in the human epidermis and keratinocyte cell lines. d. The number of genes with m6A peaks in the 3’UTR of mRNA transcripts. e. Venn diagram showing 3710 common genes with m6A peaks in the 3’UTRs from four different samples. [file 41232_2023_288_MOESM16_ESM.tif]

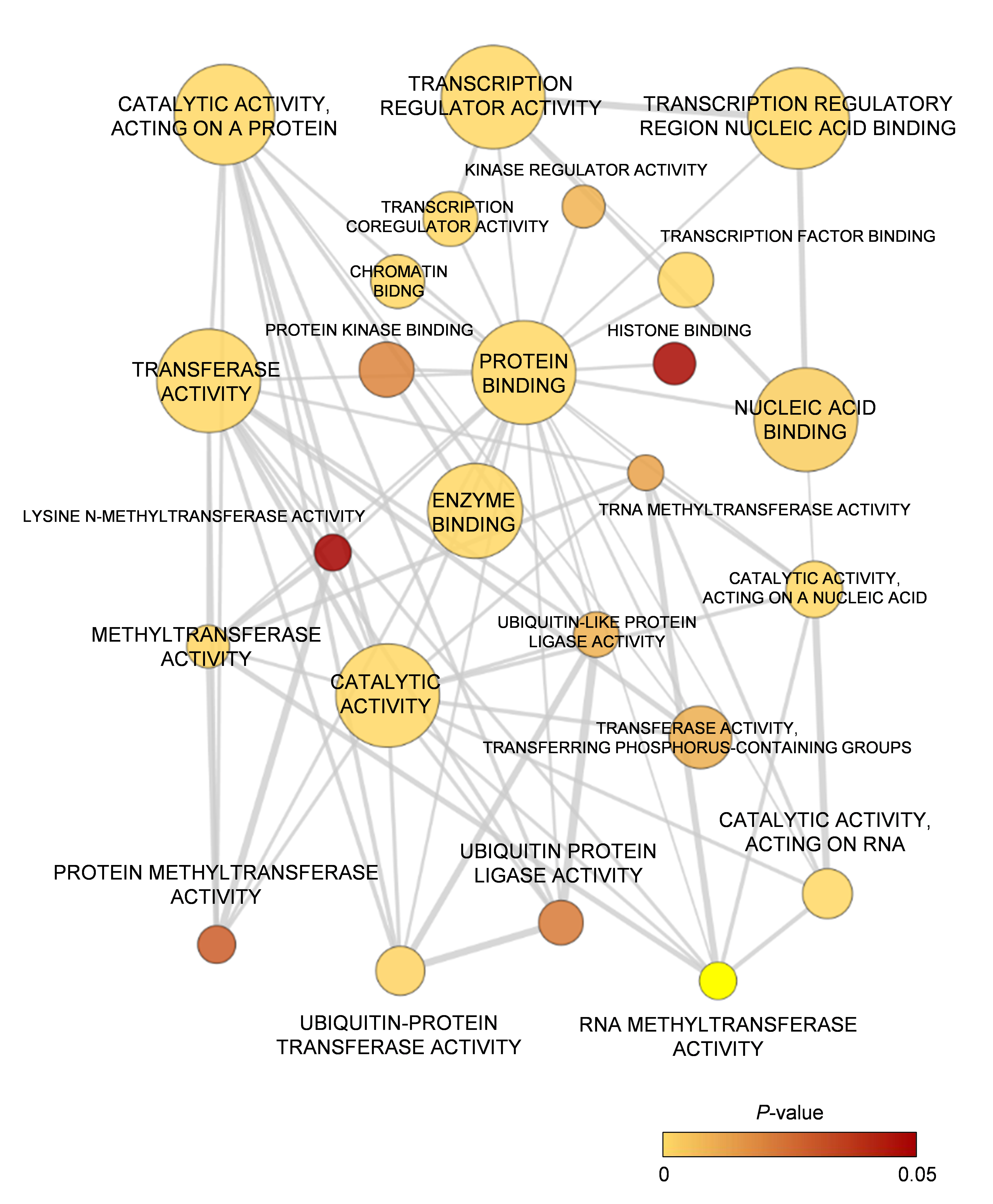

Supplement: Supplementary file 17 — Additional file 17: Fig. S9. GO enrichment map showing the molecular functions of 3710 genes with m6A peaks in the 3’UTRs of mRNA transcripts. [file 41232_2023_288_MOESM17_ESM.tif]

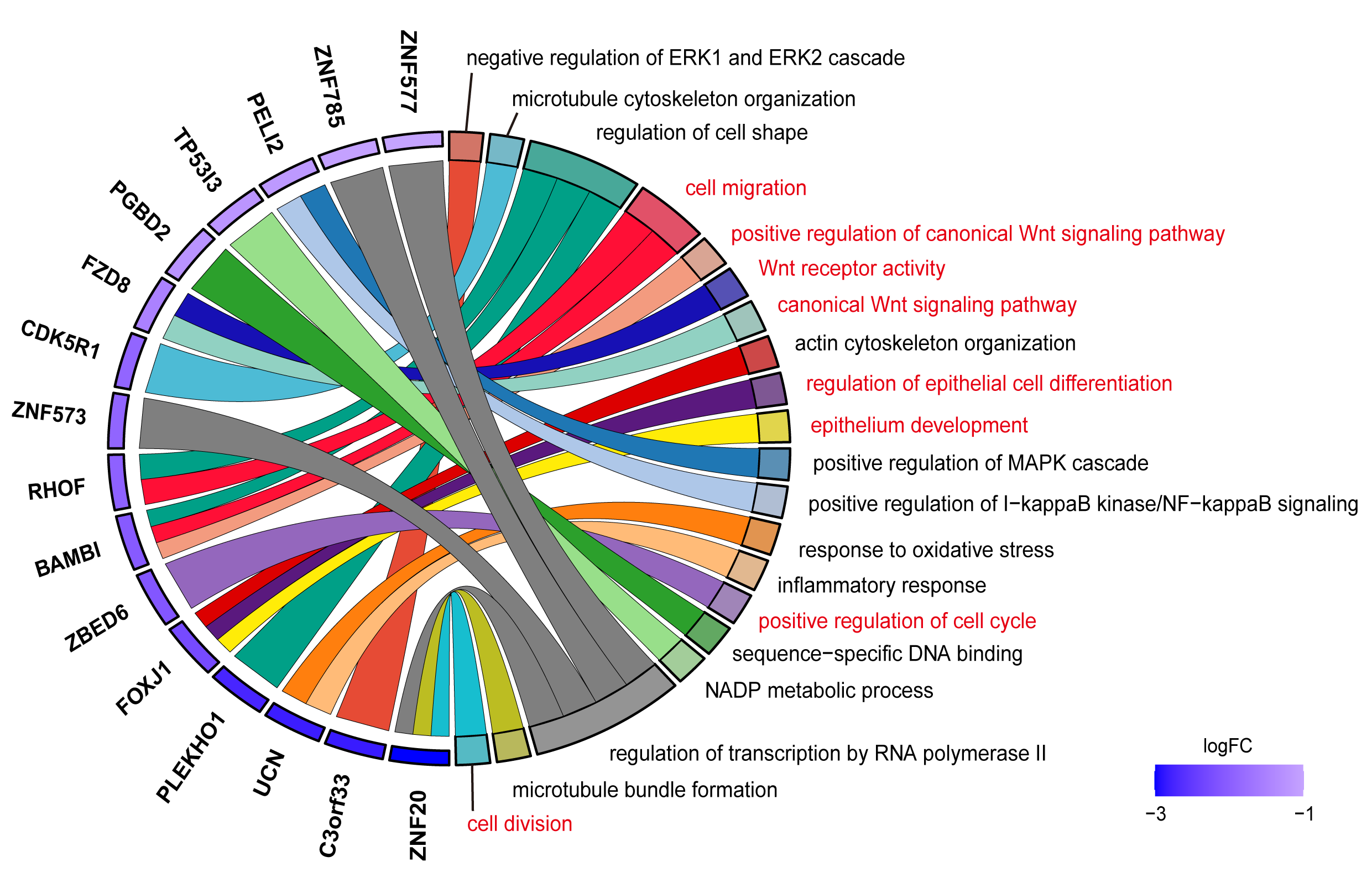

Supplement: Supplementary file 18 — Additional file 18: Fig. S10. Gene ontology analysis and a Circus plot of 16 potential target genes of ALKBH5. These genes were associated with the regulation of epithelium development; cellular functions such as epithelial cell differentiation, cell division, cell migration, and cytoskeleton organization; and signaling pathways including Wnt signaling [file 41232_2023_288_MOESM18_ESM.tif]

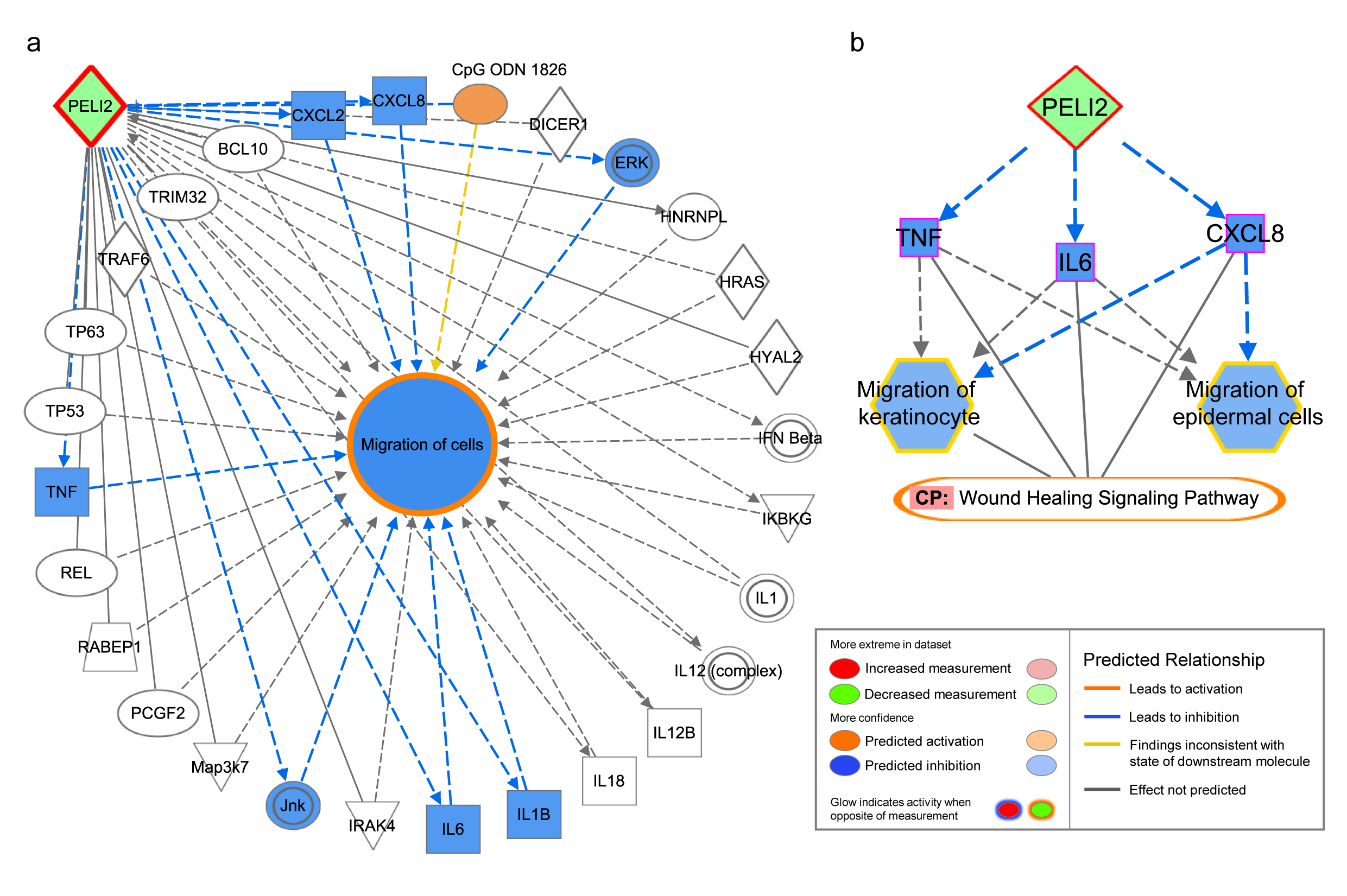

Supplement: Supplementary file 19 — Additional file 19: Fig. S11. The correlation of PELI2 with cell migration as indicated by IPA analysis. a. The molecular regulatory network by which PELI2 functions in cell migration as predicted by IPA. b. IPA predicted the regulatory network by which PELI2 functions in the migration of keratinocytes or epidermal cells and the wound healing process. [file 41232_2023_288_MOESM19_ESM.tif]

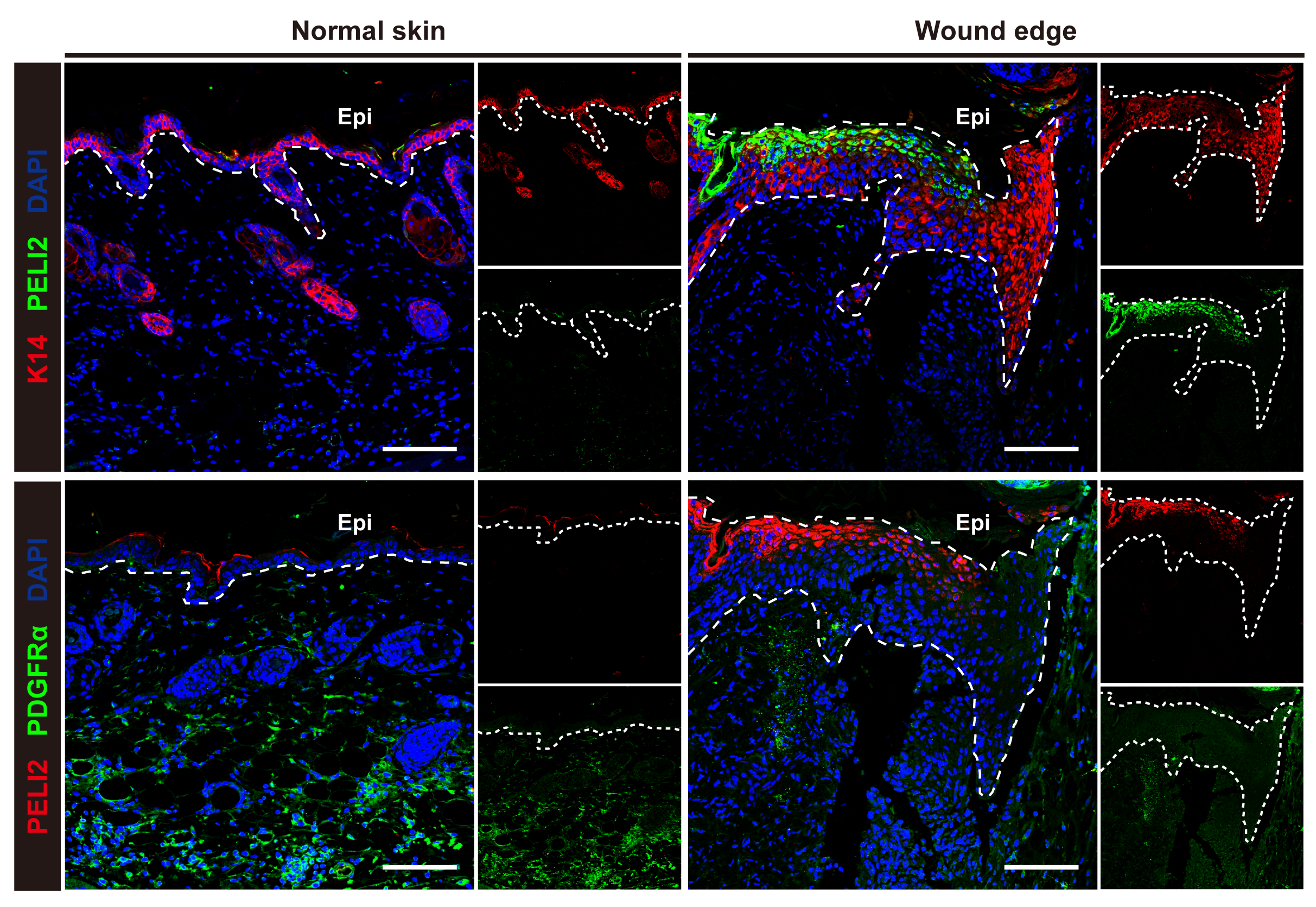

Supplement: Supplementary file 20 — Additional file 20: Fig. S12. The expression of PELI2 in keratinocytes and fibroblasts at the normal skin and wound edge. Dotted lines denote epidermal boundaries. Epi, epidermis. Scale bar: 100 μm. [file 41232_2023_288_MOESM20_ESM.tif]

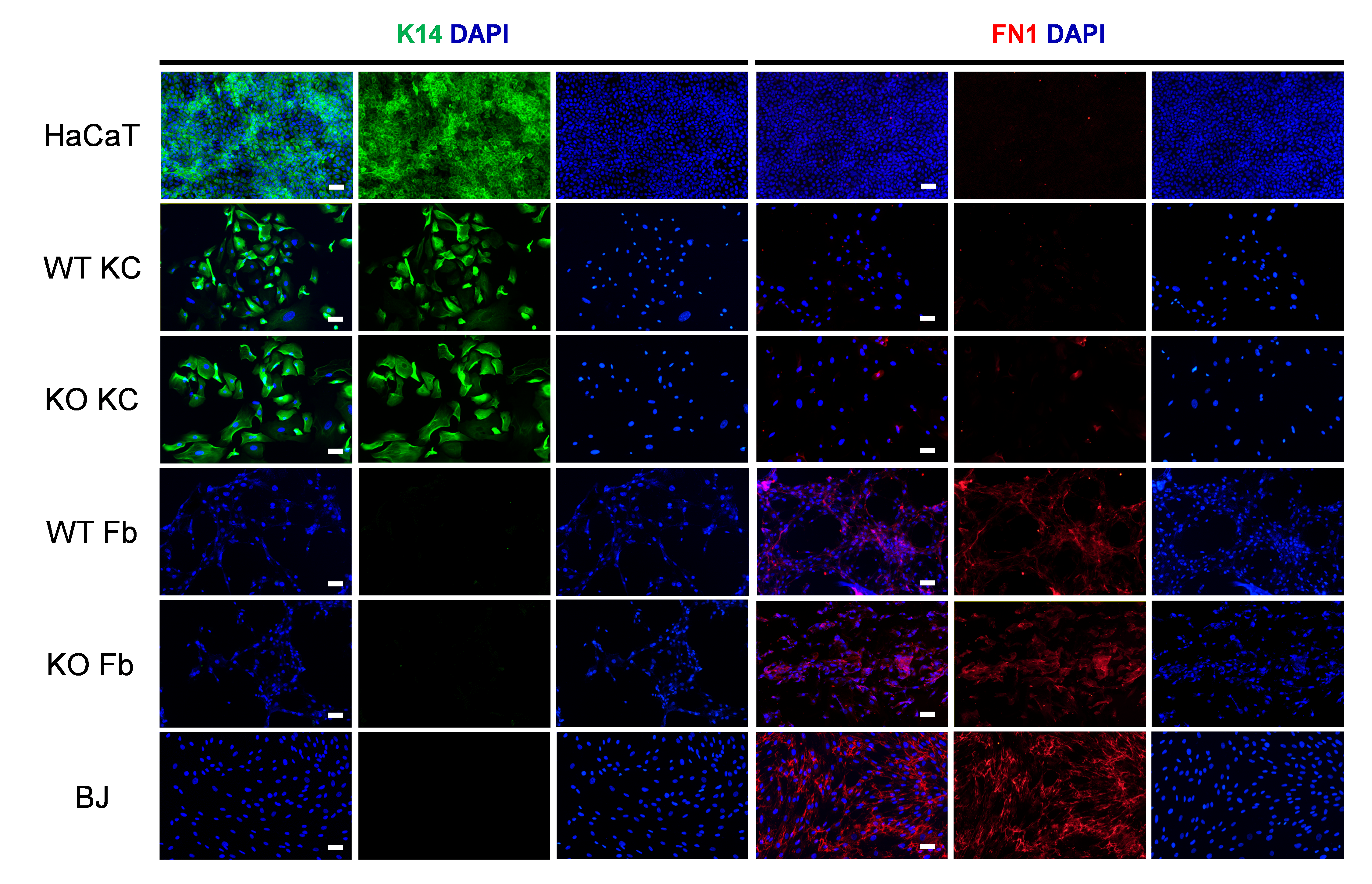

Supplement: Supplementary file 21 — Additional file 21: Fig. S13. The characterization of primary keratinocytes by IF staining of specific markers. IF showing the expression of the keratinocyte marker keratin 14 and the fibroblast marker fibronectin 1in primary keratinocytes and fibroblasts extracted from the skin of WT and Alkbh5‒/‒mice. A keratinocyte cell lineand fibroblast cell linewere used as references. Scale bar: 50 μm. KC, keratinocyte; Fb, fibroblast. [file 41232_2023_288_MOESM21_ESM.tif]
