# Supplementary material for: ALKBH5-mediated m6A demethylation fuels cutaneous wound re-epithelialization by enhancing PELI2 mRNA stability
Source: Inflamm Regen. 2023 Jul 14;43:36. doi: 10.1186/s41232-023-00288-0 (PMC10347733; doi:10.1186/s41232-023-00288-0)
Supplement: Supplementary file 15 — Additional file 15: Table S8. The total m6A peaks identified by MeRIP‒seq. [file 41232_2023_288_MOESM15_ESM.docx]

**Table S8. The total m^6^A peaks identified by MeRIP‒seq**

| Samples | HaCAT‒rep1 | HaCAT‒rep2 | Epi‒1 | Epi‒2 |
| --- | --- | --- | --- | --- |
| Total peaks | 18731 | 20548 | 19130 | 20010 |
| Cutoff value: PEAK_CUTOFF_FDR=0.05, FOLD_ENRICHMENT=1 | | | | |
